# Sociodemographic, health-related, and social predictors of subjective well-being among Chinese oldest-old: a national community-based cohort study

**DOI:** 10.1186/s12877-021-02071-7

**Published:** 2021-02-16

**Authors:** Gang Cheng, Yan Yan

**Affiliations:** Department of Epidemiology and Health Statistics, Xiangya School of Public Health, Central South University, Changsha, Hunan China

**Keywords:** Subjective well-being, Multifactor analysis, Chinese oldest-old, Large and representative sample

## Abstract

**Background:**

There is still a lack of systematic investigation of comprehensive contextual factors of subjective well-being (SWB) among Chinese oldest-old. This study aimed to explore sociodemographic, health-related, and social predictors of SWB among Chinese oldest-old using a large and representative sample.

**Methods:**

The study included 49,069 individuals aged 80 and older from the Chinese Longitudinal Healthy Longevity Survey, a prospective, nationwide, community-based study conducted from 1998 to 2014. SWB was measured by eight items covering life satisfaction, positive affect (optimism, happiness, personal control, and conscientiousness), and negative affect (anxiety, loneliness, and uselessness). Generalized estimating equation models were used to explore the predictors of SWB.

**Results:**

We found that age, gender, ethnic group, education, primary occupation before retirement, current marital status, and place of residence were sociodemographic predictors of SWB among the Chinese oldest-old. The health-related predictors included self-rated health, visual function, hearing function, diet quality, smoking status, drinking status, and exercise status. SWB was influenced by some social factors, such as the number of biological siblings, the number of children, leisure activities, financial independence, and access to adequate medical service. In particular, self-rated health, access to adequate medical services, exercise status, and place of residence exert a stronger effect than other factors.

**Conclusions:**

SWB in the oldest-old is influenced by a large number of complex sociodemographic, health-related, and social factors. Special attention should be paid to the mental health of centenarians, women, rural residents, widowed, physically disabled, and childless oldest-old people. Relevant agencies can improve physical activities, leisure activities, financial support, and medical services to promote the well-being of the oldest-old.

**Supplementary Information:**

The online version contains supplementary material available at 10.1186/s12877-021-02071-7.

## Background

The world’s population is rapidly aging. The population aged 60 years and older is expected to rise to 2 billion by 2050 [1]. People worldwide are living longer, and now the oldest-old (aged 80 and above) are the fastest-growing age group globally. The number of the oldest-old will have quadrupled between 2000 and 2050 to 434 million [1]. China is one of the fastest aging countries and has the largest oldest-old population in the world [2]. There is growing evidence that a person’s dynamic psychological aspects might be related to long-term survival [3, 4]. Substantial evidence indicates that subjective well-being (SWB) as an important predictor of longevity was associated with a decreased risk of all-cause mortality [5, 6].

There are two recognized theoretical perspectives in well-being research: SWB emphasizes hedonism or happiness, and psychological well-being (PWB) focuses on eudaemonia or self-realization [7]. Although SWB is highly correlated to PWB, they belong to different constructs in terms of positive psychological function [8]. SWB is defined as the cognitive and affective evaluations of one’s life [9]. The cognitive aspect usually refers to one’s judgments regarding life satisfaction [9, 10]. The affective aspect typically refers to one’s moods, emotions, and feelings [9], which is measured by the frequency and intensity of positive affect and negative affect [11]. In brief, SWB involves three components: life satisfaction, positive affect, and negative affect. PWB is considered to be a process of realizing values that make us feel alive and real, give us a sense of life, and seek to develop personal potential and to achieve full function [7, 12, 13]. PWB was composed of self-acceptance, autonomy, purpose in life, personal growth, positive relationships, and environmental mastery [13]. To some extent, it is difficult to distinguish between SWB and PWB. In previous studies, more focused on PWB than on SWB, and some mistook SWB for PWB [14, 15]. Our study explores the predictors of SWB from the real meaning of it.

In recent years, psychologists have paid more attention to the SWB of older people (aged 60 and above) and its related factors [16]. However, most studies focused on SWB and its related factors among older people and seldom considered those among the oldest-old [17]. Life span theories have proposed that older people can regulate age-related losses (e.g., loss of family or friends, decreased physical or cognitive functioning) and adapt their old life [18, 19], but researchers have assumed that age-related losses can severely limit this adaptability in oldest-old age [17, 20, 21]. Previous researches have found a downward trend in the SWB level with increasing age among older people [22]. ‘A common stereotype about aging’ showed that a decrease in gains and an increase in losses, such as poor health condition and physical disability, which are more common in oldest-old adults, may lead to their worse SWB [23, 24]. Previous studies have suggested that the predictors of SWB can differ across age groups [17, 25–27]. A meta-analysis suggested that the association of physical and social resources with SWB was stronger in the oldest-old than in the young-old (aged 65–79) [17, 26]. Subsequent studies pointed out that health factors played a particularly strong role in the young-old, while specific social factors became more important in the oldest-old [27]. In the previous literature, the Georgia Centenarian Study found significant direct or indirect effects of physical health impairment, social resources, cognitive functioning, and education on positive aspects among oldest-old adults [28]. Nakagawa et al. reported that cognitive function, hearing problems, and activities of daily living (ADL) were strong predictors of well-being in both Japanese and American centenarians [17]. A Spanish study suggested that SWB among the oldest-old was mainly predicted by personality traits [29]. In a Swedish study, social network quality, self-rated overall health, sense of controlling one’s life, and depression were significantly associated with life satisfaction [30]. The limitations of these existing literature are that the sample size is too small (less than 350) and not representative enough.

There have been a few studies of the SWB among the oldest-old in developed countries, but the results should not be extended to developing countries or different cultures [16]. In developed and developing economies, the relationships between the same factor and SWB may be inconsistent. For example, some researchers have reported the strongest correlations between economic status and SWB in poor developing countries but the weakest correlations in the wealthier developed countries [31]. Diener pointed out the cultural differences in SWB that SWB was influenced by the environment and social circumstances [32]. There are both universal and culture-specific predictors of well-being [32]. One theory of culture and self has proposed that individuals learn cultural values through their cultural background and internalize them into their personal beliefs [33]. Individuals in independent societies such as American and Western countries attach importance to self-esteem and personal feelings in judging SWB, whereas individuals in interdependent societies such as Asia and Eastern countries value relational harmony and social norms [33, 34].

The Chinese Longitudinal Healthy Longevity Survey (CLHLS) is a dynamic, prospective, and national cohort of Chinese older people, which is the largest longitudinal study of the oldest-old in the world [2, 16]. Some studies have obtained related results about factors of SWB using CLHLS data. Zhang et al. reported that better intergenerational relations promoted older people’s positive affect and reduced their negative affect [35]. Chen et al. found that coresidence with spouse or children was associated with positive well-being [36]. Brown et al. reported a strong negative relationship between religious participation and SWB [37]. Li et al. observed that life satisfaction and affective aspect were both influenced by demographic variables and social supports [16]. One limitation of this study is that the analysis of variance was used to examine the influences without adjusting for any confounders. To sum up, there is still a lack of systematic investigation of comprehensive contextual factors of SWB among Chinese oldest-old. Based on the bottom-up theories which explain and predict SWB by focusing on objective life circumstances [38], the present study could capture contextual factors of SWB comprehensively.

To fill the gap in the existing research literature, we explore the sociodemographic, health-related, and social predictors of SWB among oldest-old people using a large and representative population from CLHLS conducted from 1998 to 2014. The prespecified hypothesis is that the oldest-old with better health conditions and social resources will have better SWB. We hope that our research findings will contribute to developing the improvement and promotion strategies of the oldest-old’s well-being.

## Methods

### Study population

The CLHLS is a dynamic, community-based, and prospective cohort study to investigate the determinants of health and longevity of Chinese older people. It is a nationwide survey covering approximately 85% of the Chinese population. Half of the counties or cities in 22 provinces and 7 longevity areas in China are randomly selected as study sites. The CLHLS began in 1998, and follow-up visits are carried out every 2–3 years. For older people who are dead and lost to follow-up, the samples were replenished nearby according to the same sex and age. The surveys were conducted by trained interviewers at the participants’ homes with structured questionnaires. Family members, caregivers, or institutional staff were interviewed when the participants were unable to answer questions, but the subjective questions, such as self-rated life satisfaction, affective aspects, and self-rated health, were answered by participants themselves. More details can be found elsewhere [2, 39, 40]. The current study was based on seven waves of the CLHLS from 1998 to 2014. From a total of 85,905 individuals, we included 51,742 who aged ≥80 years and completed SWB assessments. Then we excluded 2673 who were diagnosed with dementia and mental disease and screened as moderate to severe cognitive impairment by a Mini-Mental State Examination score of 20 or less. Finally, a sample size of 49,069 individuals was included in our analysis (Additional file 1 Fig. S1). Excluding 18,752 individuals who were followed up repeatedly, there were 30,317 participants. According to the requirements for multiple logistics regression analysis [41], the total sample size calculated for this study was at least 3000 and this large sample study met the requirements.

### Measurements

#### Outcome

SWB was measured by eight items covering life satisfaction, positive affect (optimism, happiness, personal control, and conscientiousness), and negative affect (anxiety, loneliness, and uselessness) [16]. Life satisfaction was assessed by “how do you rate your life at present” with a five-point Likert scale ranging from 1 (very bad) to 5 (very good). For the affective aspects, participants responded to the questions “do you always look on the bright side of things?” “are you as happy as when you were younger?” “can you make your own decisions concerning your personal affairs?” “do you like to keep your belongings neat and clean?” “do you often feel fearful or anxious?” “do you often feel lonely and isolated?” “do you feel the older you get, the more useless you are?” on a five-point Likert scale ranging from 1 (always) to 5 (never). The SWB score was constructed from the sum of the eight-item scores and was divided by quartile (Q1 = 0–26, Q2 = 27–29, Q3 = 30–32, and Q4 = 33–40) [15]. We reversed the coding of negative affect, so that the higher the score, the more positive the SWB. If the score was within the range of Q4, the participants were classified as having a better SWB, if not, the participants were classified as having a worse SWB [42]. The internal consistency of the index (Cronbach’s α) was 0.68. Because only eight items were used to construct the index and the alpha was positively related to the number of items used, the index was considered reasonable [36]. Confirmatory factor analysis supported a unidimensional construct of this SWB scale (see Additional file 2), which has good construct validity to measure the SWB of the oldest-old.

#### Predictors

Based on previous research [42–44], we divide the potential predictors into three categories: sociodemographic, health-related, and social factors. The sociodemographic factors included age (80–89/90–99/≥100 years), gender (men/women), ethnic group (Han nationality/others), education (0 year/ ≥1 years), primary occupation before retirement (white-collar workers/others), current marital status (never married/married and not separated/separated/divorced/widowed), have been widowed (yes/no), and place of residence (city/town/rural areas). Health-related factors included ADL score, ADL disability (yes/no), self-rated health (very good/good/fair/bad/very bad), the number of natural teeth, visual function (blind/vision loss/normal), hearing function (deaf/hearing loss/normal), systolic blood pressure, diastolic blood pressure, pulse pressure, heart rate, self-reported diseases (yes/no), comorbidity of self-reported diseases (yes/no), food frequency score, smoking status (never/past/current), drinking status (never/past/current), and exercise status (never/past/current). Social factors included co-residence (living alone/in an institution/with family members), the number of cohabitants, the number of biological siblings, siblings at death (yes/no), the number of children, children at death (yes/no), leisure activities score, financial independence (primary financial source from retirement wages or own work/other sources), caregiver when sick (nobody/live-in caregiver/social services/friends or neighbors/family members), and access to adequate medical service (yes/no). Except for those variables that did not change over time, including gender, ethnic group, education, and primary occupation before retirement, the number of biological siblings, and the number of children, other variables were time-dependent and measured repeatedly.

ADL was measured according to whether the interviewees need any assistance in six basic daily activities (bathing, dressing, going to the toilet, indoor transfer, continence, and eating) based on the international standard of Katz’s ADL index [45]. “Without assistance”, “one part assistance”, and “more than one part assistance” were respectively scored 1, 2, and 3. These six items were summed up to ADL scores ranging from 6 to 18 and the higher scores, the more difficultly their ADL (Cronbach’s α = 0.82). The interviewees were considered as ADL disability if they answered “more than one part of assistance” in performing one of the six basic daily activities. Self-rated health was assessed by “how do you rate your health at present?’ with a five-point Likert scale ranging from 1 (very bad) to 5 (very good), thus a higher score indicated a better health status. Visual function was objectively examined by “could the interviewee see a break in the circle on the cardboard sheet when lit by a flashlight and distinguish where the break was located?”. Hearing function was measured by “was the interviewee able to hear what you said?”. After interviewees had rested for at least five minutes, interviewers used a mercury sphygmomanometer (upper arm type; Yuyue, Jiangsu, China) to measure blood pressure twice on the right arm. Korotkoff phase I was the systolic blood pressure and phase V was the diastolic blood pressure. For bedridden interviewees, blood pressure was measured in the recumbent position [46]. Pulse pressure was calculated as the difference between systolic and diastolic blood pressure. The heart rate was the number of times interviewees’ heartbeats per minute, which was tested by the interviewer with a stethoscope.

Self-reported diseases were measured by asking the interviewee “are you suffering from hypertension, diabetes, heart disease, cerebrovascular disease, respiratory disease, and cancer?”. Comorbidity of self-reported diseases means that the interviewee reported suffering from two or more diseases. The food frequency score was the sum of the frequency of eating fresh fruit, vegetables, meat, fish, eggs, and beans. “Rarely or never”, “occasionally”, and “always or often” were respectively scored 1, 2, and 3. The higher the score, the better the diet quality (Cronbach’s α = 0.60). Interviewees also reported the frequency of eight leisure activities which they participated in with a five-point Likert scale ranging from 1 (never) to 5 (always). The eight leisure activities are housework, personal outdoor activities, garden work, reading newspapers or books, raising domestic animals, playing cards, watching TV or listening to the radio, and social activities. These leisure activities were summed to the leisure activities score ranging from 8 to 40, and the higher scores indicated the more frequent leisure activities (Cronbach’s α = 0.60).

### Statistical analyses

First, descriptive statistics of the basic characteristics of 30,317 participants at the initial survey were provided. Data are expressed as counts (percentages) because they are all categorical variables. Since SWB is an ordinal categorical outcome variable, the Wilcoxon rank-sum test and Kruskal-Wallis H test were used to compare the differences in better and worse SWB by covariates. Gender differences in the basic variables were examined by chi-square tests. Second, to indirectly infer whether these predictors are possible predictors of each item, we used Kendall’s rank correlation coefficients to assess the inter-correlations among SWB and each item, because they are all ordinal categorical variables. Third, collinearity diagnosis analysis for the preditors of SWB was conducted. The absolute value of correlation coefficient > 0.5, tolerance < 0.2, variance inflation ≥5, or condition index > 30 and the variance proportion of two or more variables > 0.5 suggested the presence of multicollinearity [47]. Fourth, the ordinal logistic regression models in the generalized estimating equation method were used to estimate the effects of the potential predictors on the SWB among 49,069 individuals. The subject variable was the number of participants and the within-subject variable was the year of follow-up. We specified a robust estimator to adjust for the standard errors clustering by the participants over time. An autoregressive working correlation structure was chosen to account for the spatial correlation within participants. Odds ratio (OR) and corresponding 99% confidence intervals (CIs) quantified the extent of effects. We established three models. Model 1 was only adjusted for the year of follow-up. To control for confounding bias, model 2 included the year of follow-up and all potential predictors. To eliminate the multicollinearity between correlated variables, model 3 removed one of the variables that had multicollinearity. However, for variables that were multiple collinear with the year of follow-up, only the interactions between them were added and the variables were not removed. Overall, a small percentage of the data for the predictors were missing (1.51%), and we used multiple imputation methods to handle these missing data [48]. To minimize the likelihood of false-positive results in the large sample study, a two-tailed *p-*value < 0.01 was considered statistically significant. All statistical analyses were performed with SPSS 22.0 statistical software (IBM SPSS Inc., New York, NY, USA).

## Result

### Participant characteristics

The basic characteristics of the study participants are shown in Table 1. Almost all the participants were of Han nationality (93.8%). Approximately 70% were illiterate, only 10% were white-collar workers. Most of the participants were widowed (77.0%) and rural residence (53.9%). Participants who were octogenarians, men, Han nationality, not illiterate, white-collar workers, and living in cities had better SWB. Besides, participants who rated their health as very good had better SWB. Conversely, participants who were considered with ADL disability and reported suffering from cerebrovascular disease and respiratory disease had worse SWB. Compared to older women, older men had a higher proportion of being octogenarians, white-collar workers, and living in cities. Moreover, older men were more likely to rate their health as very good and report suffering from cerebrovascular disease and respiratory disease. However, older women were more likely to be illiterate, widowed, and considered with ADL disability. The population with missing data and those estimated by multiple imputations had similar basic characteristics (see Additional file 1 Table S1).

**Table 1:** The basic characteristics of study participants of 30,317 Chinese oldest-old people at the initial survey

Variables	Total (*n* = 30,317)	Better SWB (*n* = 6512)	Worse SWB (*n* = 23,805)	Women (*n* = 17,663)	Men (*n* = 12,654)
**Age group**					
80–89 years	13,419 (44.3)	3309 (24.7)	10,110 (75.3)**^a^	6567 (37.2)	6852 (54.1)**
90–99 years	9923 (32.7)	1968 (19.8)	7955 (80.2)	5600 (31.7)	4323 (34.2)
≥ 100 years	6975 (23.0)	1235 (17.7)	5740 (82.3)	5496 (31.1)	1479 (11.7)
**Gender**					
Women	17,663 (58.3)	3346 (18.9)	14,317 (81.1)**		
Men	12,654 (41.7)	3166 (25.0)	9488 (75.0)		
**Ethnic group**					
Han nationality	28,442 (93.8)	6250 (22.0)	22,192 (78.0)**	16,532 (93.6)	11,910 (94.1)
Ethnic minorities	1875 (6.2)	262 (14.0)	1613 (86.0)	1131 (6.4)	744 (5.9)
**Education**					
0 year	20,317 (67.0)	3691 (18.2)	16,626 (81.8)**	2375 (13.4)	7625 (60.3)**
≥ 1 years	10,000 (33.0)	2821 (28.2)	7179 (71.8)	15,288 (86.6)	5029 (39.7)
**Primary occupation before retirement**					
White-collar	2207 (7.3)	866 (39.2)	1341 (60.8)**	400 (2.3)	1807 (14.3)**
Others	28,110 (92.7)	5646 (20.1)	22,464 (79.9)	17,263 (97.7)	10,847 (85.7)
**Current marital status**					
Married and not separated	6119 (20.2)	1729 (28.3)	4390 (71.7)**	1457 (8.2)	4662 (36.8)**
Separated	412 (1.4)	77 (18.7)	335 (81.3)	106 (0.6)	306 (2.4)
Divorced	132 (0.4)	24 (18.2)	108 (81.8)	50 (0.3)	82 (0.6)
Widowed	23,332 (77.0)	4619 (19.8)	18,713 (80.2)	15,954 (90.3)	7378 (58.3)
Never married	322 (1.1)	63 (19.6)	259 (80.4)	96 (0.5)	226 (1.8)
**Place of residence**					
City	6866 (22.6)	2153 (31.4)	4713 (68.6)**^a^	3731 (21.1)	3135 (24.8)**
Town	7116 (23.5)	1513 (21.3)	5603 (78.7)	4110 (23.3)	3006 (23.8)
Rural areas	16,335 (53.9)	2846 (17.4)	13,489 (82.6)	9822 (55.6)	6513 (51.5)
**ADL disability**					
Yes	4884 (16.1)	791 (16.2)	4093 (83.8)**	3476 (19.7)	1408 (11.1)**
No	25,433 (83.9)	5721 (22.5)	19,712 (77.5)	14,187 (80.3)	11,246 (88.9)
**Self-rated health**					
Very good	3692 (12.2)	1981 (53.7)	1711 (46.3)**^a^	1946 (11.0)	1746 (13.8)**
Good	12,678 (41.8)	3020 (23.8)	9658 (76.2)	7295 (41.3)	5383 (42.5)
Fair	10,288 (33.9)	1261 (12.3)	9027 (87.7)	6135 (34.7)	4153 (32.8)
Bad	3390 (11.2)	240 (7.1)	3150 (92.9)	2128 (12.0)	1262 (10.0)
Very bad	269 (0.9)	10 (3.7)	259 (96.3)	159 (0.9)	110 (0.9)
**Hypertension**					
Yes	5252 (17.3)	1088 (20.7)	4164 (79.3)	3073 (17.4)	2179 (17.2)
No	25,065 (82.7)	5424 (21.6)	19,641 (78.4)	14,590 (82.6)	10,475 (82.8)
**Diabetes**					
Yes	541 (1.8)	135 (25.0)	406 (75.0)	290 (1.6)	251 (2.0)
No	29,776 (98.2)	6377 (21.4)	23,399 (78.6)	17,373 (98.4)	12,403 (98.0)
**Heart disease**					
Yes	2486 (8.2)	565 (22.7)	1921 (77.3)	1407 (8.0)	1079 (8.5)
No	27,831 (91.8)	5947 (21.4)	21,884 (78.6)	16,256 (92.0)	11,575 (91.5)
**Cerebrovascular disease**					
Yes	1302 (4.3)	230 (17.7)	1072 (82.3)*	635 (3.6)	667 (5.3)**
No	29,015 (95.7)	6282 (21.7)	22,733 (78.3)	17,028 (96.4)	11,987 (94.7)
**Respiratory disease**					
Yes	3593 (11.9)	696 (19.4)	2897 (80.6)*	1802 (10.2)	1791 (14.2)**
No	26,724 (88.1)	5816 (21.8)	20,908 (78.2)	15,861 (89.8)	10,863 (85.8)
**Cancer**					
Yes	124 (0.4)	30 (24.2)	94 (75.8)	64 (0.4)	60 (0.5)
No	30,193 (99.6)	6482 (21.5)	23,711 (78.5)	17,599 (99.6)	12,594 (99.5)

*Abbreviation*: *SWB* subjective well-being, *ADL* activities of daily living. Data are expressed as counts (percentages). Level of significance: * *p* **<** 0.01, ** *p* **<** 0.001. ^**a**^ There is a statistically significant difference in the pairwise comparison between this group and any other groups

### Inter-correlations between SWB, self-rated life satisfaction, and affective aspects

Table 2 shows the inter-correlations between SWB, self-rated life satisfaction, and affective aspect. Significant associations were found among the nine variables. SWB was positively correlated with self-rated life satisfaction and positive affect (optimism, happiness, personal control, and conscientiousness), but negatively correlated with negative affect (anxiety, loneliness, and uselessness). Self-rated life satisfaction and the positive affect were positively related to each other but negatively correlated with the negative affect. The negative affect was also positively related to each other.

**Table 2:** The inter-correlations between SWB, life satisfaction, positive affects and negative affects among 30,317 Chinese oldest-old people

Variables	1	2	3	4	5	6	7	8
1. SWB								
2. life satisfaction	0.330							
3. optimism	0.371	0.328						
4. happiness	0.428	0.245	0.248					
5. personal control	0.358	0.106	0.178	0.204				
6. conscientiousness	0.320	0.264	0.336	0.188	0.167			
7. anxiety	−0.407	−0.141	−0.234	−0.179	−0.145	−0.118		
8. loneliness	−0.432	−0.197	−0.241	−0.224	−0.131	−0.140	0.496	
9. uselessness	−0.389	−0.156	−0.182	−0.206	−0.085	−0.111	0.249	0.309

*Abbreviation*: *SWB* subjective well-being. Data are expressed as Kendall’s rank correlation coefficients. All the *p*-values were less than 0.001

### Predictors of better SWB

Table S2 shows the presence of multicollinearity between current marital status and having been widowed, ADL score and ADL disability, systolic blood pressure and pulse pressure, heart disease and comorbidity of self-reported diseases, and co-residence and the number of cohabitants. Table S3 and Table S4 show that there is no multicollinearity among the predictors after excluding systolic blood pressure. Table 3 shows the OR and 99% CIs of the predictors of better SWB. In the final multivariable-adjusted model (model 3), centenarians, Han nationality, receiving more than 1 year of schooling, white-collar workers, and living in cities or towns were significantly associated with a better SWB (*p* < 0.01), compared to octogenarians, ethnic minorities, illiteracy, others workers, and living in rural areas. In terms of health-related factors, rating their health as very good or good, getting higher food frequency score, smoked in the past, drinking at present, and exercising at present were significantly associated with a better SWB (*p* < 0.01), compared to rating their life satisfaction and health as fair, getting lower food frequency score, never smoking, never drinking, and never exercising. In terms of social factors, more biological siblings, more children, more frequent leisure activities, financial independence, and access to adequate medical services were significantly associated with a better SWB (*p* < 0.01), compared to fewer biological siblings, fewer children, less frequent leisure activities, financial dependence, and lack of adequate medical services. Besides, men, widowed, rating their health as bad or very bad, blind or vision loss, and deaf or hearing loss respectively reduced the odds of better SWB by 12, 16, 35% or 63, 25% or 34, and 22% or 19% (*p* < 0.01), compared to female, married and not separated, rating their health as fair, normal visual function, and normal hearing function.

**Table 3:** Odds ratio and 99% confidence intervals of the predictors of better subjective well-being among 49,069 Chinese oldest-old people

Variables	Model 1	Model 2	Model 3
**Sociodemographic factors**			
Ages 90–99 (vs. 80–89)	0.81 (0.76, 0.86)**	1.02 (0.95, 1.10)	1.03 (0.95, 1.11)
Ages ≥100 (vs. 80–89)	0.69 (0.63, 0.74)**	1.13 (1.02, 1.25)*	1.14 (1.04, 1.27)*
Men (vs. women)	1.46 (1.37, 1.55)**	0.88 (0.81, 0.96)**	0.88 (0.81, 0.96)**
Han nationality (vs. ethnic minorities)	1.68 (1.46, 1.93)**	1.49 (1.29, 1.72)**	1.50 (1.30, 1.73)**
≥ 1 years of schooling (vs. 0 year)	1.78 (1.67, 1.89)**	1.10 (1.02, 1.20)*	1.11 (1.02, 1.20)*
White-collar (vs. others)	2.58 (2.34, 2.84)**	1.28 (1.14, 1.44)**	1.28 (1.14, 1.44)**
Separated (vs. married and not separated)	0.61 (0.48, 0.79)**	0.79 (0.60, 1.04)	0.80 (0.60, 1.05)
Divorced (vs. married and not separated)	0.63 (0.40, 1.00)*	0.71 (0.42, 1.18)	0.72 (0.43, 1.19)
Widowed (vs. married and not separated)	0.64 (0.60, 0.69)**	0.79 (0.69, 0.90)**	0.84 (0.77, 0.91)**
Never married (vs. married and not separated)	0.65 (0.48, 0.89)*	0.88 (0.62, 1.25)	0.88 (0.62, 1.24)
Have been widowed (vs. no)	0.72 (0.67, 0.78)**	1.09 (0.96, 1.24)	
City (vs. rural areas)	2.25 (2.10, 2.41)**	1.55 (1.42, 1.69)**	1.56 (1.44, 1.70)**
Town (vs. rural areas)	1.32 (1.23, 1.42)**	1.16 (1.07, 1.25)**	1.16 (1.07, 1.25)**
**Heath related factors**			
ADL score	0.88 (0.87, 0.90)**	0.96 (0.93, 0.99)**	1.00 (0.98, 1.02)
ADL disability (vs. no)	0.69 (0.63, 0.75)**	1.36 (1.17, 1.57)**	
Self-rated health			
Very good (vs. fair)	8.24 (7.52, 9.03)**	6.75 (6.12, 7.44)**	6.76(6.13, 7.45)**
Good (vs. fair)	2.27(2.11, 2.44)**	2.02(1.88, 2.18)**	2.02(1.88, 2.18)**
Bad (vs. fair)	0.56(0.49, 0.64)**	0.65 (0.57, 0.75)**	0.65 (0.56, 0.74)**
Very bad (vs. fair)	0.27 (0.15, 0.46)**	0.38 (0.22, 0.67)**	0.37 (0.21, 0.66)**
Number of natural teeth	1.01 (1.01, 1.02)**	1.00 (0.99, 1.00)	1.00 (0.99, 1.00)
Blind (vs. normal)	0.44 (0.40, 0.48)**	0.75 (0.67, 0.83)**	0.75 (0.67, 0.83)**
Vision loss (vs. normal)	0.45 (0.42, 0.49)**	0.67 (0.61, 0.73)**	0.66 (0.61, 0.73)**
Deaf (vs. normal)	0.49 (0.41, 0.59)**	0.77 (0.63, 0.94)*	0.78 (0.64, 0.95)*
Hearing loss (vs. normal)	0.56 (0.52, 0.60)**	0.81 (0.75, 0.87)**	0.81 (0.75, 0.88)**
Systolic blood pressure	1.00 (1.00, 1.00)*	279.39 (0.13, 5.99 × 10^5^)	
Diastolic blood pressure	1.00 (0.99, 1.00)**	0.00 (1.66 × 10^−6^,7.65)	1.00 (0.99, 1.00)
Pulse pressure	1.00 (1.00, 1.00)	0.00 (1.68 × 10^−6^, 7.70)	1.00 (1.00, 1.01)
Heart rate	1.00 (0.99, 1.00)**	1.00 (1.00, 1.01)	1.00 (1.00, 1.01)
Hypertension (vs. no)	0.92 (0.85, 0.99)	0.91 (0.82, 1.01)	0.91 (0.82, 1.01)
Diabetes (vs. no)	1.01 (0.82, 1.24)	0.96 (0.76, 1.21)	0.95 (0.76, 1.20)
Heart disease (vs. no)	1.05 (0.95, 1.16)	1.04 (0.90, 1.19)	
Cerebrovascular disease (vs. no)	0.80 (0.70, 0.92)**	0.93 (0.79, 1.10)	0.92 (0.78, 1.09)
Respiratory disease (vs. no)	0.86 (0.79, 0.94)**	0.97 (0.87, 1.07)	0.96 (0.87, 1.07)
Cancer (vs. no)	0.97 (0.63, 1.48)	1.12 (0.718, 1.79)	1.11 (0.70, 1.77)
Comorbidity of self-reported diseases (vs. no)	0.93 (0.85, 1.03)	1.06 (0.89, 1.27)	1.09 (0.94, 1.27)
Food frequency score	1.21 (1.19, 1.22)**	1.08 (1.07, 1.10)**	1.08 (1.07, 1.10)**
Smoking at present (vs. never smoking)	1.28 (1.18, 1.38)**	1.07 (0.98, 1.18)	1.07 (0.98, 1.18)
Smoked in the past (vs. never smoking)	1.37 (1.27, 1.48)**	1.16 (1.06, 1.28)**	1.17 (1.07, 1.28)**
Drinking at present (vs. never drinking)	1.37 (1.27, 1.47)**	1.09 (1.00, 1.19)*	1.09 (1.00, 1.18)*
Drank in the past (vs. never drinking)	1.06 (0.97, 1.15)	0.97 (0.87, 1.07)	0.96 (0.87, 1.07)
Exercising at present (vs. never exercising)	2.65 (2.49, 2.82)**	1.47 (1.36, 1.58)**	1.48 (1.37, 1.59)**
Exercised in the past (vs. never exercising)	1.32 (1.20, 1.44)**	1.10 (1.00, 1.22)	1.11 (1.00, 1.23)
**Social factors**			
Living alone (vs. living with family members)	0.82 (0.75, 0.89)**	0.99 (0.89, 1.11)	
Living in an institution (vs. living with family members)	1.14 (0.99, 1.31)	1.17 (0.90, 1.52)	
Number of cohabitants	1.00 (0.98, 1.01)	1.02 (1.00, 1.04)*	1.02 (1.00, 1.03)
Number of biological siblings	1.03 (1.02, 1.05)**	1.02 (1.00, 1.04)*	1.02 (1.00, 1.04)*
Siblings at death (vs. no)	0.98 (0.90, 1.06)	0.99 (0.90, 1.10)	0.99 (0.90, 1.09)
Number of children	1.02 (1.00, 1.03)*	1.02 (1.01, 1.04)**	1.02 (1.01, 1.04)**
Children at death (vs. no)	0.84 (0.79, 0.90)**	0.97 (0.91, 1.04)	0.98 (0.91, 1.05)
Leisure activities	1.10 (1.09, 1.10)**	1.04 (1.03, 1.04)**	1.04 (1.03, 1.04)**
Financial independence (vs. no)	2.29 (2.14, 2.45)**	1.23 (1.13, 1.35)**	1.24 (1.13, 1.35)**
Caregiver when sick			
Nobody (vs. family members)	0.63 (0.50, 0.80)**	0.96 (0.72, 1.28)	0.95 (0.72, 1.27)
Live-in caregiver (vs. family members)	1.20 (0.99, 1.45)	1.02 (0.83, 1.26)	1.02 (0.83, 1.26)
Social services (vs. family members)	1.13 (0.98, 1.31)	1.18 (0.89, 1.55)	1.34 (1.14, 1.59)**
Friends or neighbors (vs. family members)	0.61 (0.41, 0.90)*	0.94 (0.60, 1.45)	0.93 (0.60, 1.45)
Access to adequate medical services (vs. no)	3.51 (3.01, 4.09)**	1.91 (1.60, 2.27)**	1.91 (1.61, 2.27)**

*Abbreviation*: *ADL* activities of daily living. Model 1 was only adjusted for the year of follow-up. Model 2 included the year of follow-up and all potential predictors. Because of multicollinearity, model 3 removed the variables of having been widowed, ADL disability, heart disease, co-residence, and systolic blood pressure. Level of significance: * p < 0.01, ** p < 0.001. Only factors that were statistically significant in all three models were considered as statistically significant predictors

## Discussion

In this large prospective community-based cohort study in China, we found that age, gender, ethnic group, education, primary occupation before retirement, current marital status, and place of residence were sociodemographic predictors of SWB among the Chinese oldest-old. Then, the health-related predictors of SWB included self-rated health, visual function, hearing function, diet quality, smoking status, drinking status, and exercise status. Moreover, SWB was influenced by some social factors, such as the number of biological siblings, the number of children, leisure activities, financial independence, and access to adequate medical service. In particular, after adjustment for possible interactions in the multivariable-adjusted model, self-rated health, access to adequate medical services, exercise status, and place of residence still exert a stronger effect on SWB among the Chinese oldest-old than other factors.

Two variables deserve attention in our study, that is, age and gender. Their promotive and inhibitory effects on SWB were reversed in unadjusted analysis and multivariate analysis. Combined with the basic characteristics (Table 1), we think that the unadjusted effect is correct. Maybe because some of the predictors are moderators of the relationships between age or gender and SWB. Consistent with an American study [24], our findings showed that relatively younger octogenarians had better mental health than centenarians. A longitudinal study demonstrated that well-being declined with impending death among the national samples from Germany, the United Kingdom, and America [49]. But Italian centenarians reported greater satisfaction with life and less anxiety and depression than younger people [50]. A general theoretical perspective revealed that the aging process, such as the declines in cognitive and physical functioning, reduced the capacity of individuals to adapt to stressful events [24, 51]. Inconsistent with previous studies in developed countries [17], we observed the gender difference in SWB among the oldest-old. Besides, there are significant gender differences in some predictors of SWB among the oldest-old. Compared to older women, older men had a higher proportion of being octogenarians and white-collar workers. Instead, older women were more likely to be illiterate and widowed. It is well known that women live longer than men. That may be why men are more likely to be octogenarian and women have lost their husbands. In traditional China, parents usually placed more emphasis on the education and development of their sons than their daughters [43]. Our research is informative to gender-tailored interventions for a better SWB among the oldest-old.

Ethnic differences have been found in life satisfaction among older people from Nepal [52], but not found in affect and loneliness among the oldest-old from American [25]. In China, the special cultural traditions, different values, and even strong religious beliefs of ethnic minorities may cause differences in SWB between ethnic minorities and Han nationality. Ethnicity also affects other key predictors of SWB, such as education, occupation, and place of residence. Among older people from Vietnam and Israel [53, 54], higher levels of education were also associated with a better SWB. But it was not found among the oldest-old from Japan, America, and Sweden [17, 25, 30]. Empirical evidence shows that education is positively related to psychological resilience, which can play a role in buffering various stressors [44, 55]. Primary occupation before retirement can reflect socioeconomic status to some extent, and it is also closely related to some predictive factors of SWB, such as financial independence, access to adequate medical service, and even diet quality. The accumulated savings, more pensions, and more social security can better support the life and psychological needs of older people. Compared to rural areas, the advantages of living in cities for better SWB are easy to explain. Residents in urban areas have easier access to better health care services and medical technologies [56], as well as a broader social network, more social activities, and more community services [43, 56]. Widowed has been proved to be a predictor of loneliness and depression among older people in China [44, 57], as well as life satisfaction among the oldest-old in Sweden [30]. The death of a spouse means the loss of some psychological support and companionship in daily life, instead of emotional sadness [44]. Our research recommended that the mental health of the widowed oldest-old need to be highly valued in the social support system.

Based on previous studies that reported the strong associations between self-rated health and one or more aspects of SWB among both the young-old and the oldest-old in developed countries [11, 30, 58], our study shows that self-rated health is a strong predictor of SWB among Chinese oldest-old. However, the association between any kind of self-reported diseases and SWB among the oldest-old was weakened and became nonsignificant. Self-rated health measures something different from physician’s ratings but depending upon one’s hypothesis, which is ‘subjective’ or ‘perceived’ as opposed to ‘objective’ or ‘actual’ [59]. Self-rated health does not decline with increasing age, to the same extent as chronic diseases and disabilities increase [59, 60]. Older people usually more positively assess their health than the middle-aged [59, 61]. Published researches have illustrated that there is a causal relationship between self-rated health and a series of psychological factors, such as self-esteem [58], depression [58, 62], and loneliness [43, 63]. The self-concept hypothesis provides evidence for the stability of self-rated health, and it reflects one’s established beliefs about their health [64, 65]. Thus, our research advocates that social services should focus more on the oldest-old’s subjective perception of their health.

Similar to related studies in developed countries [17, 66], our findings suggest that visual and hearing impairment has a significant negative impact on the SWB of the oldest-old. Visual and hearing disabilities will adversely affect the interpersonal communication and activity of the daily life of older people [67, 68]. These disabilities can also cause older people to be fearful of or anxious about the unknown world that they can’t see or hear. Our research reminds us to pay more attention to the mental health of disabled older people. Similar to our research, the previous study among Canadian older men has observed that frequent consumption of vegetables and fruit is associated with greater life satisfaction [69]. Nutrition-related health problems include frailty, depression, visual function, chronic non-communicable diseases, and so on [70]. Proper nutrition is a modifiable factor that ultimately improves health, prevents functional disability, and promotes one’s well being [69]. To some extent, smoking and drinking can indeed make people relaxed and release some psychological stress. However, the damage of smoking and drinking to physical functions, such as increased risks of lung cancer, atherosclerosis, and liver cirrhosis [71, 72], makes it not recommended. Previous researches have indicated that physical activity is a significant and robust predictor of SWB among older people in developed countries [10, 22, 73]. Our study shows that this prediction is still applicable for the oldest-old. Physical activity is linked with the release of emotion-related neurotransmitters, including norepinephrine, dopamine, serotonin, and endorphins [10, 74]. Under the condition that the oldest-old physical function permits, close relatives and social workers should help them to participate in physical activities.

Siblings relationship is almost the longest lasting in a person’s lives, which plays an important role in the social and emotional support of older people [75, 76]. There is robust evidence indicating that the sibling relationship is significantly related to depression, anxiety, loneliness, and life satisfaction among young-old adults both in developed and developing countries [75–77]. As observed in a recent paper, children are related to better SWB and lack of depression among older people in European countries [78]. The roles of children in providing instrumental, emotional, and economic support for older people make them indispensable in the later stages of one’s life. In agreement with published articles focusing on older people from Croatia, our findings showed that engagement in leisure activities contributed to better SWB [79, 80]. Through participating in leisure activities, the oldest-old can meet life values and needs, build social relationships, feel positive emotions, and therefore enhance the well-being [80].

One novel finding from our study is that the oldest-old with financial dependence more likely to face worse SWB than others with financial independence. Related research has revealed that receiving financial support from adult children considerably increased male older people’s negative aspects [35]. It was guessed that financial independence probably means autonomy, which is directly related to their SWB. Besides, for the oldest-old, their children are mostly at or near retirement age, thus they can only get little financial support from their children. One finding which needs to be taken seriously is that access to adequate medical service makes a powerful impact on the SWB of the oldest-old. Older people in remote, poor, rural areas and those left behind cannot get adequate medical service. The social phenomenon of “the difficulty in seeing a doctor” among older people is prevalent in China. Because of travel inconvenience and medical procedures cumbersome, it is difficult for older people to timely and quick access to medical services. Medical treatment may become a major mental distress for older people. Because of this, relevant departments should actively provide convenient, low-cost, and effective medical and health services for older people, especially the oldest-old.

Given the differences compared with studies in other countries, the current study suggested that gender, ethnicity, and education may be culture-specific factors. These factors are endowed with unique cultural connotations in different countries due to history or tradition. This study found that self-rated health, access to adequate medical services, exercise status, and place of residence were more strongly associated with SWB among the Chinese oldest-old than other factors. From an American study, well-being among adults aged 75 and over was found to be most influenced by friendships, spouse, and financial security, while that among adults aged 65–74 was most influenced by satisfaction with spouse, friendships, and government services [81]. Moreover, there may be consistency across age groups regarding the associations of self-rated health and sibling relationship with SWB. These comparisons may not be accurate because of inconsistencies in measurement methods and the lack of large samples of the oldest-old. Additional cross-country and cross-age researches are required to ascertain the country-specific and age-specific differences and mechanisms regarding the predictors of SWB.

There are several strengths to our study. First, to the best of our knowledge, the present study is the first to carry out a systematic investigation of comprehensive contextual factors of SWB among the oldest-old in China. Second, the current study included a large and representative sample of the Chinese oldest-old, allowing a robust assessment for the predictors of SWB among this group. Third, this article carried on a preliminary exploration of all available and possible sociodemographic, health-related, and social factors, which were obtained through face-to-face surveys. Fourth, our results supported that SWB was significantly correlated with self-rated life satisfaction, optimism, happiness, personal control, conscientiousness, anxiety, loneliness, and uselessness. It is reasonable to infer that the predictors of SWB may have a similar effect on these dimensions. Finally, our findings may also apply to other populations in transition, particularly in East Asia and Southeast Asia (e.g. Malaysia, Nepal), where contextual background and cultures are similar to those in China, such as the breakdown of traditional family structures and the imperfection of social security systems [43].

This study has several limitations. First, those strong predictors of SWB among the oldest-old may interact with other variables, and we overestimate their effects on SWB by not adjusting the interactions. Continued efforts to explore these relevant interactions further will yield more robust and realistic predictive effects. Second, during the follow-up period from 1998 to 2014, great changes have taken place in China, such as the rise of the Internet and electronic information technology, the government’s greater concern for people’s livelihood, and the improvement of people’s quality of life. These changes will more or less have an impact on the content of this study. Subsequent studies suggest that study populations with a small-time span be selected. Third, in our study, SWB was not measured by other more valid and reliable scales, such as the Philadelphia Geriatric Center Morale Scale and the Well-Being subscale of the Differential Personality Questionnaire. The Cronbach’s α of our SWB scale was only 0.68, thus the reliability of the scale was defective. Fourth, our study used a single-factor structure of the SWB indicator, which varied from previous studies that distinguished life satisfaction and affective aspects [16, 36]. The factor structure of SWB in oldest-old people should be further examined. Fifth, detection bias might exist because most information of variables came from the interviewee’s report and was not verified again. Finally, because of massive missing or unavailable data, the study did not explore other possible predictors, such as household income, community services, and personality traits. The effects of these factors can be discussed in future studies.

## Conclusions

In this cohort study of over 30,000 oldest-old people in China, we observed that SWB in the oldest-old is influenced by a large number of complex sociodemographic, health-related, and social factors, including age, gender, ethnic group, education, primary occupation before retirement, current marital status, place of residence, self-rated health, visual function, hearing function, diet quality, smoking status, drinking status, exercise status, the number of biological siblings, the number of children, leisure activities, financial independence, and access to adequate medical service. Our research advocates that special attention should be paid to the mental health of centenarians, women, rural residents, widowed, physically disabled, and childless oldest-old people. Relevant agencies can improve physical activities, leisure activities, financial support, and medical services to promote the well-being of the oldest-old. Future study is expected to explore other social factors that can be intervened, such as community services and social support.

## Supplementary Information

**Additional file 1: Fig. S1.** Flowchart of the study population. **Table S1.** The basic characteristics with missing values of study participants of 30,317 Chinese oldest-old people at the initial survey. **Table S2.** The correlation coefficients among the predictors and year of follow-up. **Table S3.** Collinearity statistics for the preditors of subjective well-being. **Table S4.** Collinearity diagnostics for the preditors of subjective well-being.

**Additional file 2.** Confirmatory factor analysis for assessing the factor structure of subjective well-being scale.

## Data Availability

The datasets analyzed during the current study are available in the [10.18170/DVN/UWS2LR ][40].

## Abbreviations

SWB: Subjective well-being
PWB: Psychological well-being
ADL: Activities of daily living
CLHLS: The Chinese Longitudinal Healthy Longevity Survey
