# Supplementary material for: Sociodemographic, health-related, and social predictors of subjective well-being among Chinese oldest-old: a national community-based cohort study
Source: BMC Geriatr. 2021 Feb 16;21:124. doi: 10.1186/s12877-021-02071-7 (PMC7885581; doi:10.1186/s12877-021-02071-7)
Supplement: Supplementary file 1 — Additional file 1: Fig. S1. Flowchart of the study population. Table S1. The basic characteristics with missing values of study participants of 30,317 Chinese oldest-old people at the initial survey. Table S2. The correlation coefficients among the predictors and year of follow-up. Table S3. Collinearity statistics for the preditors of subjective well-being. Table S4. Collinearity diagnostics for the preditors of subjective well-being. [file 12877_2021_2071_MOESM1_ESM.docx]

**Sociodemographic, health-related, and social predictors of** **subjective well-being among Chinese oldest-old: a national community-based cohort study**

Gang Cheng, Yan Yan

Department of Epidemiology and Health Statistics, Xiangya School of Public Health, Central South University, Changsha, Hunan, China

**
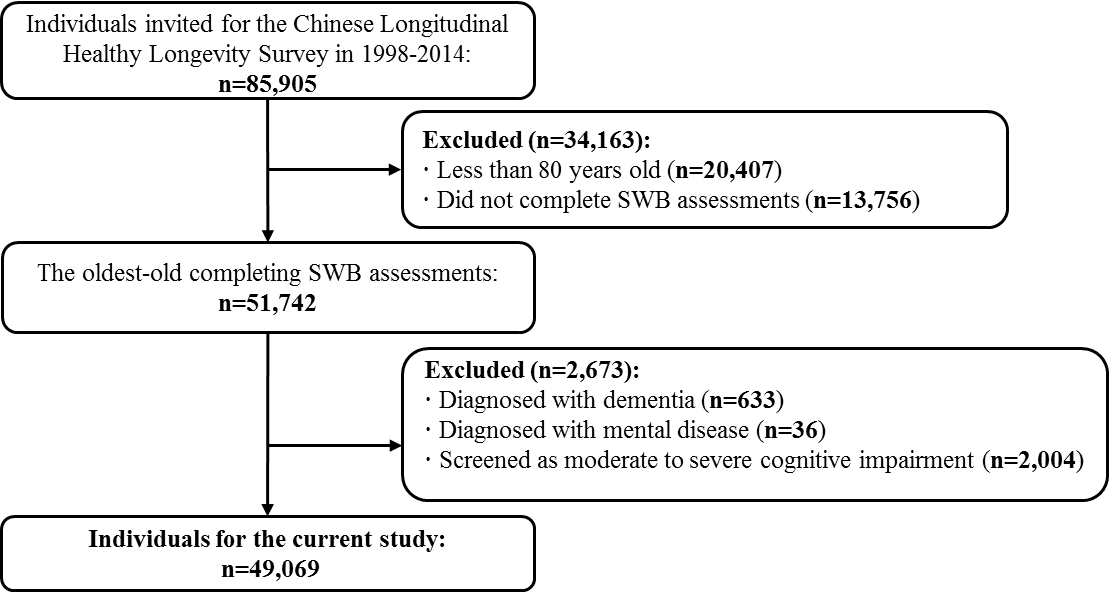
**

**Figure S1** Flowchart of the study population

**Table S1** The basic characteristics with missing values of study participants of 30,317 Chinese oldest-old people at the initial survey

| Variables | Total | Better SWB | Worse SWB | Women | Men |
| --- | --- | --- | --- | --- | --- |
| **Age group** |  |  |  |  |  |
| 80-89 years | 13419(44.3) | 3309(24.7) | 10110(75.3)****^a^** | 6567(37.2) | 6852(54.1)** |
| 90-99 years | 9923(32.7) | 1968(19.8) | 7955(80.2) | 5600(31.7) | 4323(34.2) |
| ≥100 years | 6975(23.0) | 1235(17.7) | 5740(82.3) | 5496(31.1) | 1479(11.7) |
| **Gender** |  |  |  |  |  |
| Women | 17663(58.3) | 3346(18.9) | 14317(81.1)** |  |  |
| Men | 12654(41.7) | 3166(25.0) | 9488(75.0) |  |  |
| **Ethnic group** |  |  |  |  |  |
| Han nationality | 28201(93.0) | 6204(22.0) | 21997(78.0)** | 16391(93.6) | 11810(94.1) |
| Ethnic minorities | 1850(6.1) | 259(14.0) | 1591(86.0) | 1114(6.4) | 736(5.9) |
| Missing | 266(0.9) |  |  |  |  |
| **Education** |  |  |  |  |  |
| 0 year | 20185(66.6) | 3663(18.1) | 16522(81.9)** | 2344(13.4) | 7604(60.4)** |
| ≥1 years | 9948(32.8) | 2806(28.2) | 7142(71.8) | 15199(86.6) | 4986(39.6) |
| Missing | 184(0.6) |  |  |  |  |
| **Primary occupation before retirement** | | |  |  |  |
| White-collar | 2179(7.2) | 856(39.3) | 1323(60.7)** | 390(2.2) | 1789(14.4)** |
| Others | 27645(91.2) | 5545(20.1) | 22100(79.9) | 17010(97.8) | 10635(85.6) |
| Missing | 493(1.6) |  |  |  |  |
| **Current marital status** | |  |  |  |  |
| Married and not  separated | 6114(20.2) | 1728(28.3) | 4386(71.7)** | 1456(8.2) | 4658(36.8)** |
| Separated | 412(1.4) | 77(18.7) | 335(81.3) | 106(0.6) | 306(2.4) |
| Divorced | 132(0.4) | 24(18.2) | 108(81.8) | 50(0.3) | 82(0.6) |
| Widowed | 23327(76.9) | 4619(19.8) | 18708(80.2) | 15950(90.3) | 7377(58.3) |

**Table S1** The basic characteristics with missing values of study participants of 30,317 Chinese oldest-old people at the initial survey (*continued*)

| Variables | Total | Better SWB | Worse SWB | Women | Men |
| --- | --- | --- | --- | --- | --- |
| Never married | 322(1.1) | 63(19.6) | 259(80.4) | 96(0.5) | 226(1.8) |
| Missing | 10(0.0) |  |  |  |  |
| **Place of residence** |  |  |  |  |  |
| City | 5256(17.3) | 1748(33.3) | 3508(66.7)****^a^** | 2960(21.6) | 2296(23.9)** |
| Town | 5260(17.4) | 1225(23.3) | 4035(76.7) | 3074(22.4) | 2186(22.8) |
| Rural areas | 12799(42.2) | 2417(18.9) | 10382(81.1) | 7694(56.0) | 5105(53.2) |
| Missing | 7002(23.1) |  |  |  |  |
| **ADL disability** |  |  |  |  |  |
| Yes | 4858(16.0) | 785(16.2) | 4073(83.8)** | 3459(19.6) | 1399(11.1)** |
| No | 25419(83.8) | 5718(22.5) | 19701(77.5) | 14181(80.4) | 11238(88.9) |
| Missing | 40(0.1) |  |  |  |  |
| **Self-rated health** |  |  |  |  |  |
| Very good | 3692(12.2) | 1981(53.7) | 1711(46.3)****^a^** | 1946(11.0) | 1746(13.8)** |
| Good | 12677(41.8) | 3020(23.8) | 9657(76.2) | 7294(41.3) | 5383(42.5) |
| Fair | 10287(33.9) | 1261(12.3) | 9026(87.7) | 6135(34.7) | 4152(32.8) |
| Bad | 3389(11.2) | 240(7.1) | 3149(92.9) | 2127(12.0) | 1262(10.0) |
| Very bad | 269(0.9) | 10(3.7) | 259(96.3) | 159(0.9) | 110(0.9) |
| Missing | 3(0.0) |  |  |  |  |
| **Hypertension** |  |  |  |  |  |
| Yes | 4966(16.4) | 1042(21.0) | 3924(79.0) | 2901(17.3) | 2065(17.1) |
| No | 23886(78.8) | 5237(21.9) | 18649(78.1) | 13895(82.7) | 9991(82.9) |
| Missing | 1465(4.8) |  |  |  |  |
| **Diabetes** |  |  |  |  |  |
| Yes | 500(1.6) | 128(25.6) | 372(74.4) | 264(1.6) | 236(2.0) |
| No | 28281(93.3) | 6154(21.8) | 22127(78.2) | 16488(98.4) | 11793(98.0) |

**Table S1** The basic characteristics with missing values of study participants of 30,317 Chinese oldest-old people at the initial survey (*continued*)

| Variables | Total | Better SWB | Worse SWB | Women | Men |
| --- | --- | --- | --- | --- | --- |
| Missing | 1536(5.1) |  |  |  |  |
| **Heart disease** |  |  |  |  |  |
| Yes | 2309(7.6) | 533(23.1) | 1776(76.9) | 1306(7.8) | 1003(8.3) |
| No | 26545(87.6) | 5747(21.7) | 20798(78.3) | 15502(92.2) | 11043(91.7) |
| Missing | 1463(4.8) |  |  |  |  |
| **Cerebrovascular disease** | |  |  |  |  |
| Yes | 1230(4.1) | 223(18.1) | 1007(81.9)* | 591(3.5) | 639(5.3)** |
| No | 27677(91.3) | 6058(21.9) | 21619(78.1) | 16252(96.5) | 11425(94.7) |
| Missing | 1410(4.7) |  |  |  |  |
| **Respiratory disease** | |  |  |  |  |
| Yes | 3436(11.3) | 675(19.6) | 2761(80.4)* | 1715(10.2) | 1721(14.2)** |
| No | 25535(84.2) | 5623(22.0) | 19912(78.0) | 15157(89.8) | 10378(85.8) |
| Missing | 1346(4.4) |  |  |  |  |
| **Cancer** |  |  |  |  |  |
| Yes | 114(0.4) | 27(23.7) | 87(76.3) | 57(0.3) | 57(0.5) |
| No | 28502(94.0) | 6207(21.8) | 22295(78.2) | 16614(99.7) | 11888(99.5) |
| Missing | 1701(5.6) |  |  |  |  |

Abbreviation: SWB, subjective well-being; ADL, activities of daily living. Data are expressed as counts (percentages). Level of significance: * *p* **<** 0.01, ** *p* **<** 0.001. **^a^** There is a statistically significant difference in the pairwise comparison between this group and any other groups.

**Table S2** The correlation coefficients among the predictors and year of follow-up

| Variables | *X*_0_ | *X*_1_ | *X*_2_ | *X*_3_ | *X*_4_ | *X*_5_ | *X*_6_ | *X*_7_ | *X*_8_ | *X*_9_ | *X*_10_ |
| --- | --- | --- | --- | --- | --- | --- | --- | --- | --- | --- | --- |
| *X*_0_ | 1 | -0.002 | -0.006 | -0.008 | -0.029^*^ | -0.031^*^ | -0.040^*^ | -0.098^*^ | -0.095^*^ | -0.052^*^ | -0.001 |
| *X*_1_ | -0.002 | 1 | -0.192^*^ | -0.024^*^ | -0.181^*^ | -0.103^*^ | 0.285^*^ | 0.282^*^ | -0.039^*^ | 0.279^*^ | 0.233^*^ |
| *X*_2_ | -0.006 | -0.192^*^ | 1 | 0.022^*^ | 0.498^*^ | 0.232^*^ | -0.351^*^ | -0.321^*^ | 0.042^*^ | -0.109^*^ | -0.100^*^ |
| *X*_3_ | -0.008 | -0.024^*^ | 0.022^*^ | 1 | 0.039^*^ | 0.040^*^ | -0.028^*^ | -0.025^*^ | 0.080^*^ | 0.041^*^ | 0.048^*^ |
| *X*_4_ | -0.029^*^ | -0.181^*^ | 0.498^*^ | 0.039^*^ | 1 | 0.328^*^ | -0.223^*^ | -0.198^*^ | 0.177^*^ | -0.070^*^ | -0.056^*^ |
| *X*_5_ | -0.031^*^ | -0.103^*^ | 0.232^*^ | 0.040^*^ | 0.328^*^ | 1 | -0.159^*^ | -0.129^*^ | 0.224^*^ | -0.014^*^ | -0.008 |
| *X*_6_ | -0.040^*^ | 0.285^*^ | -0.351^*^ | -0.028^*^ | -0.223^*^ | -0.159^*^ | 1 | **0.765^*^** | -0.046^*^ | 0.090^*^ | 0.088^*^ |
| *X*_7_ | -0.098^*^ | 0.282^*^ | -0.321^*^ | -0.025^*^ | -0.198^*^ | -0.129^*^ | **0.765^*^** | 1 | -0.038^*^ | 0.091^*^ | 0.086^*^ |
| *X*_8_ | -0.095^*^ | -0.039^*^ | 0.042^*^ | 0.080^*^ | 0.177^*^ | 0.224^*^ | -0.046^*^ | -0.038^*^ | 1 | 0.058^*^ | 0.081^*^ |
| *X*_9_ | -0.052^*^ | 0.279^*^ | -0.109^*^ | 0.041^*^ | -0.070^*^ | -0.014^*^ | 0.090^*^ | 0.091^*^ | 0.058^*^ | 1 | **0.804**^*^ |
| *X*_10_ | -0.001 | 0.233^*^ | -0.100^*^ | 0.048^*^ | -0.056^*^ | -0.008 | 0.088^*^ | 0.086^*^ | 0.081^*^ | **0.804^*^** | 1 |
| *X*_11_ | -0.078^*^ | -0.010^*^ | 0.039^*^ | 0.028^*^ | 0.038^*^ | 0.029^*^ | -0.009 | 0.005 | 0.042^*^ | -0.120^*^ | -0.091^*^ |
| *X*_12_ | 0.001 | -0.246^*^ | 0.140^*^ | -0.005 | 0.111^*^ | 0.096^*^ | -0.153^*^ | -0.136^*^ | 0.028^*^ | -0.113^*^ | -0.111^*^ |
| *X*_13_ | 0.150^*^ | 0.242^*^ | -0.143^*^ | 0.005 | -0.149^*^ | -0.086^*^ | 0.114^*^ | 0.094^*^ | -0.067^*^ | 0.258^*^ | 0.215^*^ |
| *X*_14_ | 0.008 | 0.294^*^ | -0.086^*^ | 0.037^*^ | -0.119^*^ | -0.068^*^ | 0.120^*^ | 0.113^*^ | -0.052^*^ | 0.246^*^ | 0.218^*^ |
| *X*_15_ | -0.107^*^ | -0.065^*^ | -0.013^*^ | 0.012^*^ | 0.010 | 0.001 | -0.005 | -0.003 | -0.017^*^ | -0.033^*^ | -0.030^*^ |
| *X*_16_ | -0.135^*^ | -0.017^*^ | -0.006 | 0.013^*^ | -0.006 | -0.021^*^ | 0.020^*^ | 0.027^*^ | -0.003 | 0.014^*^ | 0.004 |
| *X*_17_ | -0.038^*^ | -0.065^*^ | -0.011 | 0.005 | 0.015^*^ | 0.015^*^ | -0.019^*^ | -0.022^*^ | -0.017^*^ | -0.047^*^ | -0.037^*^ |
| *X*_18_ | -0.043^*^ | 0.001 | -0.047^*^ | -0.028^*^ | -0.025^*^ | -0.017^*^ | 0.019^*^ | 0.011^*^ | -0.016^*^ | 0.002 | -0.001 |
| *X*_19_ | 0.112^*^ | -0.094^*^ | -0.010 | 0.036^*^ | 0.041^*^ | 0.054^*^ | -0.034^*^ | -0.044^*^ | 0.039^*^ | 0.012^*^ | 0.006 |
| *X*_20_ | 0.051^*^ | -0.050^*^ | 0.012^*^ | 0.022^*^ | 0.040^*^ | 0.070^*^ | -0.042^*^ | -0.039^*^ | 0.063^*^ | 0.035^*^ | 0.029^*^ |
| *X*_21_ | 0.037^*^ | -0.062^*^ | 0.008 | 0.016^*^ | 0.070^*^ | 0.105^*^ | -0.048^*^ | -0.049^*^ | 0.124^*^ | 0.041^*^ | 0.054^*^ |
| *X*_22_ | 0.058^*^ | -0.056^*^ | 0.042^*^ | 0.022^*^ | 0.041^*^ | 0.056^*^ | -0.053^*^ | -0.048^*^ | 0.045^*^ | 0.154^*^ | 0.121^*^ |
| *X*_23_ | -0.013^*^ | -0.051^*^ | 0.063^*^ | 0.006 | 0.047^*^ | 0.034^*^ | -0.044^*^ | -0.038^*^ | 0.033^*^ | 0.036^*^ | 0.032^*^ |
| *X*_24_ | 0.010^*^ | -0.006 | 0.011 | 0.012^*^ | 0.017^*^ | 0.025^*^ | -0.013^*^ | -0.011^*^ | 0.023^*^ | 0.038^*^ | 0.033^*^ |
| *X*_25_ | 0.078^*^ | -0.084^*^ | 0.025^*^ | 0.024^*^ | 0.071^*^ | 0.096^*^ | -0.062^*^ | -0.062^*^ | 0.090^*^ | 0.077^*^ | 0.071^*^ |
| *X*_26_ | 0.009 | -0.009^*^ | 0.066^*^ | 0.017^*^ | 0.143^*^ | 0.148^*^ | -0.050^*^ | -0.031^*^ | 0.182^*^ | -0.022^*^ | 0.010 |
| *X*_27_ | -0.041 | -0.154^*^ | 0.436^*^ | 0.037^*^ | 0.237^*^ | 0.068^*^ | -0.161^*^ | -0.151^*^ | 0.004 | -0.086^*^ | -0.072^*^ |
| *X*_28_ | -0.073^*^ | -0.061^*^ | 0.302^*^ | -0.003 | 0.151^*^ | 0.050^*^ | -0.090^*^ | -0.078^*^ | -0.013^*^ | -0.071^*^ | -0.066^*^ |
| *X*_29_ | -0.038^*^ | -0.128^*^ | 0.189^*^ | 0.068^*^ | 0.229^*^ | 0.198^*^ | -0.098^*^ | -0.080^*^ | 0.216^*^ | -0.142^*^ | -0.101^*^ |
| *X*_30_ | 0.051^*^ | -0.069^*^ | -0.027^*^ | 0.025^*^ | -0.017^*^ | -0.020^*^ | 0.214^*^ | 0.127^*^ | -0.028^*^ | -0.099^*^ | -0.094^*^ |
| *X*_31_ | -0.104^*^ | 0.095^*^ | -0.040^*^ | -0.088^*^ | -0.054^*^ | -0.068^*^ | 0.052^*^ | 0.071^*^ | -0.083^*^ | 0.040^*^ | 0.031^*^ |
| *X*_32_ | 0.004 | -0.026^*^ | 0.003 | -0.015^*^ | 0.021^*^ | 0.012^*^ | -0.020^*^ | -0.016^*^ | -0.001 | -0.021^*^ | -0.010 |
| *X*_33_ | -0.005 | 0.091^*^ | -0.022^*^ | -0.017^*^ | -0.024^*^ | -0.021^*^ | 0.043^*^ | 0.045^*^ | -0.029^*^ | 0.019^*^ | 0.023^*^ |
| *X*_34_ | 0.000 | 0.004 | -0.038^*^ | -0.053^*^ | -0.010^*^ | -0.042^*^ | -0.039^*^ | 0.010^*^ | -0.043^*^ | 0.002 | 0.015^*^ |
| *X*_35_ | -0.121^*^ | 0.210^*^ | -0.160^*^ | -0.068^*^ | -0.107^*^ | -0.078^*^ | 0.143^*^ | 0.159^*^ | -0.037^*^ | 0.055^*^ | 0.052^*^ |
| *X*_36_ | 0.091^*^ | -0.358^*^ | 0.205^*^ | 0.019^*^ | 0.261^*^ | 0.208^*^ | -0.228^*^ | -0.207^*^ | 0.110^*^ | -0.366^*^ | -0.318^*^ |
| *X*_37_ | -0.012^*^ | -0.197^*^ | 0.322^*^ | 0.051^*^ | 0.349^*^ | 0.411^*^ | -0.243^*^ | -0.192^*^ | 0.337^*^ | -0.051^*^ | -0.029^*^ |
| *X*_38_ | -0.041^*^ | -0.028^*^ | 0.003 | 0.025^*^ | 0.042^*^ | 0.079^*^ | 0.087^*^ | 0.027^*^ | 0.103^*^ | 0.038^*^ | 0.021^*^ |
| *X*_39_ | -0.012 | -0.016^*^ | 0.037^*^ | 0.026^*^ | 0.055^*^ | 0.050^*^ | -0.035^*^ | -0.026^*^ | 0.082^*^ | -0.032^*^ | -0.011 |

**Table S2** The correlation coefficients among the predictors and year of follow-up (*continued*)

| Variables | *X*_11_ | *X*_12_ | *X*_13_ | *X*_14_ | *X*_15_ | *X*_16_ | *X*_17_ | *X*_18_ | *X*_19_ | *X*_20_ |
| --- | --- | --- | --- | --- | --- | --- | --- | --- | --- | --- |
| *X*_0_ | -0.078^*^ | 0.001 | 0.150^*^ | 0.008 | -0.107^*^ | -0.135^*^ | -0.038^*^ | -0.043^*^ | 0.112^*^ | 0.051^*^ |
| *X*_1_ | -0.010^*^ | -0.246^*^ | 0.242^*^ | 0.294^*^ | -0.065^*^ | -0.017^*^ | -0.065^*^ | 0.001 | -0.094^*^ | -0.050^*^ |
| *X*_2_ | 0.039^*^ | 0.140^*^ | -0.143^*^ | -0.086^*^ | -0.013^*^ | -0.006 | -0.011 | -0.047^*^ | -0.010 | 0.012^*^ |
| *X*_3_ | 0.028^*^ | -0.005 | 0.005 | 0.037^*^ | 0.012^*^ | 0.013^*^ | 0.005 | -0.028^*^ | 0.036^*^ | 0.022^*^ |
| *X*_4_ | 0.038^*^ | 0.111^*^ | -0.149^*^ | -0.119^*^ | 0.010 | -0.006 | 0.015^*^ | -0.025^*^ | 0.041^*^ | 0.040^*^ |
| *X*_5_ | 0.029^*^ | 0.096^*^ | -0.086^*^ | -0.068^*^ | 0.001 | -0.021^*^ | 0.015^*^ | -0.017^*^ | 0.054^*^ | 0.070^*^ |
| *X*_6_ | -0.009 | -0.153^*^ | 0.114^*^ | 0.120^*^ | -0.005 | 0.020^*^ | -0.019^*^ | 0.019^*^ | -0.034^*^ | -0.042^*^ |
| *X*_7_ | 0.005 | -0.136^*^ | 0.094^*^ | 0.113^*^ | -0.003 | 0.027^*^ | -0.022^*^ | 0.011 | -0.044^*^ | -0.039^*^ |
| *X*_8_ | 0.042^*^ | 0.028^*^ | -0.067^*^ | -0.052^*^ | -0.017^*^ | -0.003 | -0.017^*^ | -0.016^*^ | 0.039^*^ | 0.063^*^ |
| *X*_9_ | -0.120^*^ | -0.113^*^ | 0.258^*^ | 0.246^*^ | -0.033^*^ | 0.014^*^ | -0.047^*^ | 0.002 | 0.012^*^ | 0.035^*^ |
| *X*_10_ | -0.091^*^ | -0.111^*^ | 0.215^*^ | 0.218^*^ | -0.030^*^ | 0.004 | -0.037^*^ | -0.001 | 0.006 | 0.029^*^ |
| *X*_11_ | 1 | 0.004 | -0.147^*^ | -0.085^*^ | 0.004 | -0.003 | 0.009 | -0.007 | -0.067^*^ | -0.040^*^ |
| *X*_12_ | 0.004 | 1 | -0.120^*^ | -0.138^*^ | 0.003 | 0.014^*^ | -0.006 | -0.006 | 0.045^*^ | 0.027^*^ |
| *X*_13_ | -0.147^*^ | -0.120^*^ | 1 | 0.275^*^ | -0.043^*^ | 0.009 | -0.054^*^ | -0.008 | 0.008 | 0.015^*^ |
| *X*_14_ | -0.085^*^ | -0.138^*^ | 0.275^*^ | 1 | -0.030^*^ | 0.006 | -0.038^*^ | -0.005 | -0.028^*^ | -0.012^*^ |
| *X*_15_ | 0.004 | 0.003 | -0.043^*^ | -0.030^*^ | 1 | 0.482^*^ | **0.814^*^** | 0.113^*^ | 0.407^*^ | 0.025^*^ |
| *X*_16_ | -0.003 | 0.014^*^ | 0.009 | 0.006 | 0.482^*^ | 1 | -0.117^*^ | 0.118^*^ | 0.259^*^ | 0.010 |
| *X*_17_ | 0.009 | -0.006 | -0.054^*^ | -0.038^*^ | **0.814^*^** | -0.117^*^ | 1 | 0.051^*^ | 0.290^*^ | 0.022^*^ |
| *X*_18_ | -0.007 | -0.006 | -0.008 | -0.005 | 0.113^*^ | 0.118^*^ | 0.051^*^ | 1 | 0.035^*^ | 0.004 |
| *X*_19_ | -0.067^*^ | 0.045^*^ | 0.008 | -0.028^*^ | 0.407^*^ | 0.259^*^ | 0.290^*^ | 0.035^*^ | 1 | 0.121^*^ |
| *X*_20_ | -0.040^*^ | 0.027^*^ | 0.015^*^ | -0.012^*^ | 0.025^*^ | 0.010^*^ | 0.022^*^ | 0.004 | 0.121^*^ | 1 |
| *X*_21_ | -0.075^*^ | 0.023^*^ | -0.003 | -0.019^*^ | 0.053^*^ | 0.029^*^ | 0.041^*^ | 0.036^*^ | 0.207^*^ | 0.129^*^ |
| *X*_22_ | -0.065^*^ | 0.017^*^ | 0.008 | 0.003 | 0.038^*^ | 0.030^*^ | 0.023^*^ | -0.016^*^ | 0.129^*^ | 0.070^*^ |
| *X*_23_ | -0.081^*^ | 0.000 | -0.006 | 0.000 | -0.009^*^ | -0.003 | -0.008 | 0.029^*^ | 0.025^*^ | 0.033^*^ |
| *X*_24_ | -0.022^*^ | -0.004 | 0.003 | 0.008 | -0.005 | -0.015^*^ | 0.005 | -0.001 | 0.006 | 0.031^*^ |
| *X*_25_ | -0.086^*^ | 0.028^*^ | 0.004 | -0.017^*^ | 0.157^*^ | 0.101^*^ | 0.111^*^ | 0.029^*^ | 0.480^*^ | 0.306^*^ |
| *X*_26_ | 0.132^*^ | 0.055^*^ | -0.083^*^ | -0.049^*^ | -0.039^*^ | 0.012^*^ | -0.052^*^ | -0.026^*^ | 0.000 | 0.026^*^ |
| *X*_27_ | 0.029^*^ | 0.057^*^ | -0.097^*^ | -0.056^*^ | -0.009 | -0.013^*^ | -0.001 | -0.017^*^ | -0.024^*^ | 0.001 |
| *X*_28_ | 0.042^*^ | 0.063^*^ | -0.077^*^ | -0.033^*^ | -0.007 | -0.002 | -0.007 | -0.008 | -0.045^*^ | -0.016^*^ |
| *X*_29_ | 0.123^*^ | 0.082^*^ | -0.156^*^ | -0.116^*^ | 0.002 | -0.002 | 0.003 | -0.020^*^ | 0.028^*^ | 0.029^*^ |
| *X*_30_ | -0.028^*^ | 0.027^*^ | -0.005 | -0.050^*^ | 0.032^*^ | 0.009^*^ | 0.031^*^ | 0.009 | 0.032^*^ | 0.002 |
| *X*_31_ | 0.027^*^ | -0.047^*^ | 0.014^*^ | 0.051^*^ | -0.008 | 0.014^*^ | -0.018^*^ | 0.011 | -0.041^*^ | -0.032^*^ |
| *X*_32_ | 0.007 | 0.004 | -0.042^*^ | -0.031^*^ | 0.023^*^ | 0.006 | 0.023^*^ | 0.010 | 0.009 | -0.006 |
| *X*_33_ | 0.001 | -0.053^*^ | 0.001 | 0.022^*^ | 0.009^*^ | -0.004 | 0.013^*^ | 0.005 | -0.009 | -0.022^*^ |
| *X*_34_ | 0.009 | -0.013^*^ | -0.021^*^ | -0.002 | 0.008 | 0.002 | 0.008 | 0.017^*^ | -0.009 | -0.004 |
| *X*_35_ | -0.013 | -0.092^*^ | 0.042^*^ | 0.065^*^ | -0.003 | 0.006 | -0.008 | 0.027^*^ | -0.050^*^ | -0.021^*^ |
| *X*_36_ | 0.157^*^ | 0.194^*^ | -0.321^*^ | -0.276^*^ | -0.006 | -0.023^*^ | 0.009 | -0.030^*^ | 0.034^*^ | 0.034^*^ |
| *X*_37_ | 0.044^*^ | 0.133^*^ | -0.135^*^ | -0.125^*^ | 0.000 | -0.019^*^ | 0.013^*^ | -0.025^*^ | 0.063^*^ | 0.073^*^ |
| *X*_38_ | -0.028^*^ | 0.024^*^ | -0.001 | -0.031^*^ | 0.007 | 0.002 | 0.007 | 0.001 | 0.021^*^ | 0.029^*^ |
| *X*_39_ | 0.100^*^ | 0.011 | -0.082^*^ | -0.044^*^ | 0.022^*^ | -0.017^*^ | 0.036^*^ | 0.011 | 0.002 | 0.008 |

**Table S2** The correlation coefficients among the predictors and year of follow-up (*continued*)

| Variables | *X*_21_ | *X*_22_ | *X*_23_ | *X*_24_ | *X*_25_ | *X*_26_ | *X*_27_ | *X*_28_ | *X*_29_ | *X*_30_ |
| --- | --- | --- | --- | --- | --- | --- | --- | --- | --- | --- |
| *X*_0_ | 0.037^*^ | 0.058^*^ | -0.013^*^ | 0.010^*^ | 0.078^*^ | 0.009 | -0.041 | -0.073^*^ | -0.038^*^ | 0.051^*^ |
| *X*_1_ | -0.062^*^ | -0.056^*^ | -0.051^*^ | -0.006 | -0.084^*^ | -0.009 | -0.154^*^ | -0.061^*^ | -0.128^*^ | -0.069^*^ |
| *X*_2_ | 0.008 | 0.042^*^ | 0.063^*^ | 0.011 | 0.025^*^ | 0.066^*^ | 0.436^*^ | 0.302^*^ | 0.189^*^ | -0.027^*^ |
| *X*_3_ | 0.016^*^ | 0.022^*^ | 0.006 | 0.012^*^ | 0.024^*^ | 0.017^*^ | 0.037^*^ | -0.003 | 0.068^*^ | 0.025^*^ |
| *X*_4_ | 0.070^*^ | 0.041^*^ | 0.047^*^ | 0.017^*^ | 0.071^*^ | 0.143^*^ | 0.237^*^ | 0.151^*^ | 0.229^*^ | -0.017^*^ |
| *X*_5_ | 0.105^*^ | 0.056^*^ | 0.034^*^ | 0.025^*^ | 0.096^*^ | 0.148^*^ | 0.068^*^ | 0.050^*^ | 0.198^*^ | -0.020^*^ |
| *X*_6_ | -0.048^*^ | -0.053^*^ | -0.044^*^ | -0.013^*^ | -0.062^*^ | -0.050^*^ | -0.161^*^ | -0.090^*^ | -0.098^*^ | 0.214^*^ |
| *X*_7_ | -0.049^*^ | -0.048^*^ | -0.038^*^ | -0.011^*^ | -0.062^*^ | -0.031^*^ | -0.151^*^ | -0.078^*^ | -0.080^*^ | 0.127^*^ |
| *X*_8_ | 0.124^*^ | 0.045^*^ | 0.033^*^ | 0.023^*^ | 0.090^*^ | 0.182^*^ | 0.004 | -0.013^*^ | 0.216^*^ | -0.028^*^ |
| *X*_9_ | 0.041^*^ | 0.154^*^ | 0.036^*^ | 0.038^*^ | 0.077^*^ | -0.022^*^ | -0.086^*^ | -0.071^*^ | -0.142^*^ | -0.099^*^ |
| *X*_10_ | 0.054^*^ | 0.121^*^ | 0.032^*^ | 0.033^*^ | 0.071^*^ | 0.010^*^ | -0.072^*^ | -0.066^*^ | -0.101^*^ | -0.094^*^ |
| *X*_11_ | -0.075^*^ | -0.065^*^ | -0.081^*^ | -0.022^*^ | -0.086^*^ | 0.132^*^ | 0.029^*^ | 0.042^*^ | 0.123^*^ | -0.028^*^ |
| *X*_12_ | 0.023^*^ | 0.017^*^ | 0.000 | -0.004 | 0.028^*^ | 0.055^*^ | 0.057^*^ | 0.063^*^ | 0.082^*^ | 0.027^*^ |
| *X*_13_ | -0.003 | 0.008 | -0.006 | 0.003 | 0.004 | -0.083^*^ | -0.097^*^ | -0.077^*^ | -0.156^*^ | -0.005 |
| *X*_14_ | -0.019^*^ | 0.003 | 0.000 | 0.008 | -0.017^*^ | -0.049^*^ | -0.056^*^ | -0.033^*^ | -0.116^*^ | -0.050^*^ |
| *X*_15_ | 0.053^*^ | 0.038^*^ | -0.009 | -0.005 | 0.157^*^ | -0.039^*^ | -0.009 | -0.007 | 0.002 | 0.032^*^ |
| *X*_16_ | 0.029^*^ | 0.030^*^ | -0.003 | -0.015^*^ | 0.101^*^ | 0.012^*^ | -0.013^*^ | -0.002 | -0.002 | 0.009 |
| *X*_17_ | 0.041^*^ | 0.023^*^ | -0.008 | 0.005 | 0.111^*^ | -0.052^*^ | -0.001 | -0.007 | 0.003 | 0.031^*^ |
| *X*_18_ | 0.036^*^ | -0.016^*^ | 0.029^*^ | -0.001 | 0.029^*^ | -0.026^*^ | -0.017^*^ | -0.008 | -0.020^*^ | 0.009 |
| *X*_19_ | 0.207^*^ | 0.129^*^ | 0.025^*^ | 0.006 | 0.480^*^ | 0.000 | -0.024^*^ | -0.045^*^ | 0.028^*^ | 0.032^*^ |
| *X*_20_ | 0.129^*^ | 0.070^*^ | 0.033^*^ | 0.031^*^ | 0.306^*^ | 0.026^*^ | 0.001 | -0.016^*^ | 0.029^*^ | 0.002 |
| *X*_21_ | 1 | 0.107^*^ | 0.101^*^ | 0.026^*^ | **0.567^*^** | 0.032^*^ | -0.006 | -0.031^*^ | 0.050^*^ | -0.013^*^ |
| *X*_22_ | 0.107^*^ | 1 | 0.030^*^ | 0.013^*^ | 0.371^*^ | 0.013^*^ | 0.006 | -0.013^*^ | 0.012^*^ | -0.019^*^ |
| *X*_23_ | 0.101^*^ | 0.030^*^ | 1 | 0.011 | 0.320^*^ | -0.012 | 0.065^*^ | 0.023^*^ | 0.021^*^ | -0.019^*^ |
| *X*_24_ | 0.026^*^ | 0.013^*^ | 0.011 | 1 | 0.088^*^ | 0.008 | 0.009 | 0.007 | 0.001 | -0.002 |
| *X*_25_ | **0.567^*^** | 0.371^*^ | 0.320^*^ | 0.088^*^ | 1 | 0.022^*^ | 0.006 | -0.028^*^ | 0.034^*^ | -0.011 |
| *X*_26_ | 0.032^*^ | 0.013^*^ | -0.012 | 0.008 | 0.022^*^ | 1 | 0.004 | 0.037^*^ | 0.171^*^ | -0.122^*^ |
| *X*_27_ | -0.006 | 0.006 | 0.065^*^ | 0.009 | 0.006 | 0.004 | 1 | 0.344^*^ | 0.107^*^ | -0.006 |
| *X*_28_ | -0.031^*^ | -0.013^*^ | 0.023^*^ | 0.007 | -0.028^*^ | 0.037^*^ | 0.344^*^ | 1 | 0.088^*^ | -0.018^*^ |
| *X*_29_ | 0.050^*^ | 0.012^*^ | 0.021^*^ | 0.001 | 0.034^*^ | 0.171^*^ | 0.107^*^ | 0.088^*^ | 1 | -0.007 |
| *X*_30_ | -0.013^*^ | -0.019^*^ | -0.019^*^ | -0.002 | -0.011 | -0.122^*^ | -0.006 | -0.018^*^ | -0.007 | 1 |
| *X*_31_ | -0.035^*^ | -0.021^*^ | 0.000 | -0.010^*^ | -0.030^*^ | 0.044^*^ | -0.016^*^ | 0.014^*^ | -0.038^*^ | **-0.520^*^** |
| *X*_32_ | 0.012^*^ | 0.007 | 0.004 | 0.000 | 0.003 | 0.010^*^ | 0.009^*^ | 0.019^*^ | 0.036^*^ | -0.018^*^ |
| *X*_33_ | -0.007 | -0.003 | -0.006 | -0.002 | -0.016^*^ | -0.002 | -0.015^*^ | 0.001 | -0.019^*^ | -0.024^*^ |
| *X*_34_ | -0.001 | -0.004 | -0.003 | -0.005 | -0.007 | 0.018^*^ | -0.025^*^ | 0.024^*^ | -0.011 | -0.056^*^ |
| *X*_35_ | -0.034^*^ | -0.043^*^ | -0.005 | -0.006 | -0.047^*^ | -0.031^*^ | -0.064^*^ | -0.002 | -0.062^*^ | -0.034^*^ |
| *X*_36_ | 0.036^*^ | -0.029^*^ | 0.007 | 0.002 | 0.023^*^ | 0.190^*^ | 0.129^*^ | 0.101^*^ | 0.347^*^ | 0.079^*^ |
| *X*_37_ | 0.129^*^ | 0.072^*^ | 0.044^*^ | 0.027^*^ | 0.116^*^ | 0.163^*^ | 0.122^*^ | 0.080^*^ | 0.256^*^ | -0.003 |
| *X*_38_ | 0.024^*^ | 0.017^*^ | 0.003 | 0.003 | 0.026^*^ | -0.042^*^ | 0.014^*^ | -0.010 | 0.039^*^ | 0.282^*^ |
| *X*_39_ | 0.011 | 0.000 | -0.010 | 0.006 | 0.006 | 0.194^*^ | 0.010^*^ | 0.025^*^ | 0.080^*^ | -0.096^*^ |

**Table S2** The correlation coefficients among the predictors and year of follow-up (*continued*)

| Variables | *X*_31_ | *X*_32_ | *X*_33_ | *X*_34_ | *X*_35_ | *X*_36_ | *X*_37_ | *X*_38_ | *X*_39_ |
| --- | --- | --- | --- | --- | --- | --- | --- | --- | --- |
| *X*_0_ | -0.104 *X*_31_ | 0.004 | -0.005 | 0.000 | -0.121^*^ | 0.091^*^ | -0.012^*^ | -0.041^*^ | -0.012 |
| *X*_1_ | 0.095^*^ | -0.026^*^ | 0.091^*^ | 0.004 | 0.210^*^ | -0.358^*^ | -0.197^*^ | -0.028^*^ | -0.016^*^ |
| *X*_2_ | -0.040^*^ | 0.003 | -0.022^*^ | -0.038^*^ | -0.160^*^ | 0.205^*^ | 0.322^*^ | 0.003 | 0.037^*^ |
| *X*_3_ | -0.088^*^ | -0.015^*^ | -0.017^*^ | -0.053^*^ | -0.068^*^ | 0.019^*^ | 0.051^*^ | 0.025^*^ | 0.026^*^ |
| *X*_4_ | -0.054^*^ | 0.021^*^ | -0.024^*^ | -0.010^*^ | -0.107^*^ | 0.261^*^ | 0.349^*^ | 0.042^*^ | 0.055^*^ |
| *X*_5_ | -0.068^*^ | 0.012^*^ | -0.021^*^ | -0.042^*^ | -0.078^*^ | 0.208^*^ | 0.411^*^ | 0.079^*^ | 0.050^*^ |
| *X*_6_ | 0.052^*^ | -0.020^*^ | 0.043^*^ | -0.039^*^ | 0.143^*^ | -0.228^*^ | -0.243^*^ | 0.087^*^ | -0.035^*^ |
| *X*_7_ | 0.071^*^ | -0.016^*^ | 0.045^*^ | 0.010 | 0.159^*^ | -0.207^*^ | -0.192^*^ | 0.027^*^ | -0.026^*^ |
| *X*_8_ | -0.083^*^ | -0.001 | -0.029^*^ | -0.043^*^ | -0.037^*^ | 0.110^*^ | 0.337^*^ | 0.103^*^ | 0.082^*^ |
| *X*_9_ | 0.040^*^ | -0.021^*^ | 0.019^*^ | 0.002 | 0.055^*^ | -0.366^*^ | -0.051^*^ | 0.038^*^ | -0.032^*^ |
| *X*_10_ | 0.031^*^ | -0.010^*^ | 0.023^*^ | 0.015^*^ | 0.052^*^ | -0.318^*^ | -0.029^*^ | 0.021^*^ | -0.011 |
| *X*_11_ | 0.027^*^ | 0.007 | 0.001 | 0.009 | -0.013^*^ | 0.157^*^ | 0.044^*^ | -0.028^*^ | 0.100^*^ |
| *X*_12_ | -0.047^*^ | 0.004 | -0.053^*^ | -0.013^*^ | -0.092^*^ | 0.194^*^ | 0.133^*^ | 0.024^*^ | 0.011 |
| *X*_13_ | 0.014^*^ | -0.042^*^ | 0.001 | -0.021^*^ | 0.042^*^ | -0.321^*^ | -0.135^*^ | -0.001 | -0.082^*^ |
| *X*_14_ | 0.051^*^ | -0.031^*^ | 0.022^*^ | -0.002 | 0.065^*^ | -0.276^*^ | -0.125^*^ | -0.031^*^ | -0.044^*^ |
| *X*_15_ | -0.008 | 0.023^*^ | 0.009 | 0.008 | -0.003 | -0.006 | 0.000 | 0.007 | 0.022^*^ |
| *X*_16_ | 0.014^*^ | 0.006 | -0.004 | 0.002 | 0.006 | -0.023^*^ | -0.019^*^ | 0.002 | -0.017^*^ |
| *X*_17_ | -0.018^*^ | 0.023^*^ | 0.013^*^ | 0.008 | -0.008 | 0.009 | 0.013^*^ | 0.007 | 0.036^*^ |
| *X*_18_ | 0.011 | 0.010 | 0.005 | 0.017^*^ | 0.027^*^ | -0.030^*^ | -0.025^*^ | 0.001 | 0.011 |
| *X*_19_ | -0.041^*^ | 0.009 | -0.009^*^ | -0.009 | -0.050^*^ | 0.034^*^ | 0.063^*^ | 0.021^*^ | 0.002 |
| *X*_20_ | -0.032^*^ | -0.006 | -0.022^*^ | -0.004 | -0.021^*^ | 0.034^*^ | 0.073^*^ | 0.029^*^ | 0.008 |
| *X*_21_ | -0.035^*^ | 0.012^*^ | -0.007 | -0.001 | -0.034^*^ | 0.036^*^ | 0.129^*^ | 0.024^*^ | 0.011 |
| *X*_22_ | -0.021^*^ | 0.007 | -0.003 | -0.004 | -0.043^*^ | -0.029^*^ | 0.072^*^ | 0.017^*^ | 0.000 |
| *X*_23_ | 0.000 | 0.004 | -0.006 | -0.003 | -0.005 | 0.007 | 0.044^*^ | 0.003 | -0.010^*^ |
| *X*_24_ | -0.010 | 0.000 | -0.002 | -0.005 | -0.006 | 0.002 | 0.027^*^ | 0.003 | 0.006 |
| *X*_25_ | -0.030^*^ | 0.003 | -0.016^*^ | -0.007 | -0.047^*^ | 0.023^*^ | 0.116^*^ | 0.026^*^ | 0.006 |
| *X*_26_ | 0.044^*^ | 0.010 | -0.002 | 0.018^*^ | -0.031^*^ | 0.190^*^ | 0.163^*^ | -0.042^*^ | 0.194^*^ |
| *X*_27_ | -0.016^*^ | 0.009 | -0.015^*^ | -0.025^*^ | -0.064^*^ | 0.129^*^ | 0.122^*^ | 0.014^*^ | 0.010^*^ |
| *X*_28_ | 0.014^*^ | 0.019^*^ | 0.001 | 0.024^*^ | -0.002 | 0.101^*^ | 0.080^*^ | -0.010^*^ | 0.025^*^ |
| *X*_29_ | -0.038^*^ | 0.036^*^ | -0.019^*^ | -0.011^*^ | -0.062^*^ | 0.347^*^ | 0.256^*^ | 0.039^*^ | 0.080^*^ |
| *X*_30_ | **-0.520**^*^ | -0.018^*^ | -0.024^*^ | -0.056^*^ | -0.034^*^ | 0.079^*^ | -0.003 | 0.282^*^ | -0.096^*^ |
| *X*_31_ | 1 | 0.035^*^ | 0.045^*^ | 0.075^*^ | 0.083^*^ | -0.106^*^ | -0.129^*^ | -0.240^*^ | 0.064^*^ |
| *X*_32_ | 0.035^*^ | 1 | 0.453^*^ | 0.104^*^ | 0.062^*^ | 0.036^*^ | 0.014^*^ | -0.019^*^ | 0.020^*^ |
| *X*_33_ | 0.045^*^ | 0.453^*^ | 1 | 0.074^*^ | 0.087^*^ | -0.044^*^ | -0.046^*^ | -0.037^*^ | 0.008 |
| *X*_34_ | 0.075^*^ | 0.104^*^ | 0.074^*^ | 1 | 0.299^*^ | 0.008 | -0.045^*^ | -0.143^*^ | 0.042^*^ |
| *X*_35_ | 0.083^*^ | 0.062^*^ | 0.087^*^ | 0.299^*^ | 1 | -0.104^*^ | -0.116^*^ | -0.037^*^ | -0.029^*^ |
| *X*_36_ | -0.106^*^ | 0.036^*^ | -0.044^*^ | 0.008 | -0.104^*^ | 1 | 0.290^*^ | 0.004 | 0.066^*^ |
| *X*_37_ | -0.129^*^ | 0.014^*^ | -0.046^*^ | -0.045^*^ | -0.116^*^ | 0.290^*^ | 1 | 0.084^*^ | 0.057^*^ |
| *X*_38_ | -0.240^*^ | -0.019^*^ | -0.037^*^ | -0.143^*^ | -0.037^*^ | 0.004 | 0.084^*^ | 1 | -0.134^*^ |
| *X*_39_ | 0.064^*^ | 0.020^*^ | 0.008 | 0.042^*^ | -0.029^*^ | 0.066^*^ | 0.057^*^ | -0.134^*^ | 1 |

Variables: *X*_0_, year of follow-up; *X*_1_, age; *X*_2_, gender; *X*_3_, ethnic group; *X*_4_, education; *X*_5_, primary occupation before retirement; *X*_6_, current marital status; *X*_7_, have been widowed; *X*_8_, place of residence; *X*_9_, activities of daily living score; *X*_10_, activities of daily living disability; *X*_11_, self-rated health; *X*_12_, number of natural teeth; *X*_13_, visual function; *X*_14_, hearing function; *X*_15_, systolic blood pressure; *X*_16_, diastolic blood pressure; *X*_17_, pulse pressure; *X*_18_, heart rate; *X*_19_, hypertension; *X*_20_, diabetes; *X*_21_, heart disease; *X*_22_, cerebrovascular disease; *X*_23_, respiratory disease; *X*_24_, cancer; *X*_25_, comorbidity of self-reported diseases; *X*_26_, food frequency score; *X*_27_, smoking status; *X*_28_, drinking status; *X*_29_, exercise status; *X*_30_, co-residence; *X*_31_, the number of cohabitants; *X*_32_, the number of biological siblings; *X*_33_, siblings at death; *X*_34_, the number of children; *X*_35_, children at death; *X*_36_, leisure activities score; *X*_37_, financial independence; *X*_38_, caregiver when sick; *X*_39_, access to adequate medical service. Level of significance: ^*^ *p* **<** 0.01. The possible presence of multicollinearity has been highlighted using bold text.

**Table S3** Collinearity statistics for the preditors of subjective well-being

| Variables | Standardized Coefficients | *t* | *p*-value | Tolerance | Variance inflation |
| --- | --- | --- | --- | --- | --- |
| (Constant) |  | 19.451 | 0.000 |  |  |
| Year of follow-up | 0.046 | 10.120 | 0.000 | 0.843 | 1.187 |
| Age | 0.025 | 5.012 | 0.000 | 0.708 | 1.412 |
| Gender | -0.018 | -3.207 | 0.001 | 0.548 | 1.824 |
| Ethnic group | 0.028 | 6.556 | 0.000 | 0.970 | 1.031 |
| Education | 0.015 | 2.944 | 0.003 | 0.660 | 1.515 |
| Primary occupation before retirement | 0.034 | 7.064 | 0.000 | 0.769 | 1.301 |
| Current marital status | -0.026 | -3.760 | 0.000 | 0.366 | 2.734 |
| Have been widowed | 0.009 | 1.361 | 0.173 | 0.400 | 2.503 |
| Place of residence | 0.072 | 15.451 | 0.000 | 0.801 | 1.249 |
| Activities of daily living score | -0.026 | -3.515 | 0.000 | 0.326 | 3.067 |
| Activities of daily living disability | 0.035 | 4.910 | 0.000 | 0.346 | 2.887 |
| Self-rated health | 0.198 | 45.898 | 0.000 | 0.935 | 1.070 |
| Number of natural teeth | -0.005 | -1.039 | 0.299 | 0.908 | 1.102 |
| Visual function | -0.047 | -9.952 | 0.000 | 0.794 | 1.260 |
| Hearing function | -0.032 | -6.909 | 0.000 | 0.833 | 1.200 |
| Systolic blood pressure | 53.113 | 1.764 | 0.078 | **1.921**×**10^-8^** | **5.205**×**10^7^** |
| Diastolic blood pressure | -0.011 | -2.522 | 0.012 | 0.844 | 1.184 |
| Pulse pressure | 0.005 | 1.145 | 0.252 | 0.850 | 1.176 |
| Heart rate | -0.003 | -0.612 | 0.540 | 0.974 | 1.027 |
| Hypertension | -0.013 | -2.393 | 0.017 | 0.605 | 1.653 |
| Diabetes | -0.007 | -1.515 | 0.130 | 0.882 | 1.134 |
| Heart disease | -0.005 | -1.017 | 0.309 | 0.627 | 1.595 |
| Cerebrovascular disease | -0.012 | -2.476 | 0.013 | 0.793 | 1.260 |
| Respiratory disease | -0.010 | -2.097 | 0.036 | 0.827 | 1.209 |
| Cancer | -0.003 | -0.657 | 0.511 | 0.985 | 1.016 |
| Comorbidity of self-reported diseases | 0.004 | 0.580 | 0.562 | 0.362 | 2.762 |
| Food frequency score | 0.079 | 17.622 | 0.000 | 0.866 | 1.154 |
| Smoking status | 0.009 | 1.856 | 0.063 | 0.749 | 1.336 |
| Drinking status | 0.012 | 2.708 | 0.007 | 0.840 | 1.191 |
| Exercise status | 0.080 | 17.228 | 0.000 | 0.805 | 1.242 |
| Co-residence | -0.005 | -0.872 | 0.383 | 0.620 | 1.614 |
| Number of cohabitants | 0.010 | 1.908 | 0.056 | 0.667 | 1.498 |
| Number of biological siblings | 0.013 | 2.688 | 0.007 | 0.781 | 1.281 |
| Siblings at death | -0.002 | -0.320 | 0.749 | 0.779 | 1.283 |
| Number of children | 0.015 | 3.455 | 0.001 | 0.873 | 1.145 |
| Children at death | -0.006 | -1.248 | 0.212 | 0.828 | 1.208 |
| Leisure activities Score | 0.110 | 20.661 | 0.000 | 0.611 | 1.636 |
| Financial Independence | 0.039 | 7.484 | 0.000 | 0.657 | 1.522 |
| Caregiver when sick | 0.003 | 0.674 | 0.500 | 0.856 | 1.168 |
| Access to adequate medical service | 0.039 | 8.926 | 0.000 | 0.928 | 1.078 |

**Table S4** Collinearity diagnostics for the preditors of subjective well-being

| Dimension | Eigenvalue | Condition index |  | Variance proportions | | | | | | | | | |
| --- | --- | --- | --- | --- | --- | --- | --- | --- | --- | --- | --- | --- | --- |
|  |  |  | (Constant) | | *X*_0_ | *X*_1_ | *X*_2_ | *X*_3_ | *X*_4_ | *X*_5_ | *X*_6_ | *X*_7_ | *X*_8_ |
| 1 | 24.678 | 1.000 | 0.00 | | 0.00 | 0.00 | 0.00 | 0.00 | 0.00 | 0.00 | 0.00 | 0.00 | 0.00 |
| 2 | 2.071 | 3.452 | 0.00 | | 0.00 | 0.00 | 0.00 | 0.00 | 0.01 | 0.03 | 0.00 | 0.00 | 0.00 |
| 3 | 1.611 | 3.914 | 0.00 | | 0.00 | 0.00 | 0.02 | 0.00 | 0.03 | 0.07 | 0.00 | 0.00 | 0.00 |
| 4 | 1.007 | 4.951 | 0.00 | | 0.00 | 0.00 | 0.00 | 0.00 | 0.00 | 0.02 | 0.00 | 0.00 | 0.00 |
| 5 | 0.966 | 5.054 | 0.00 | | 0.00 | 0.00 | 0.00 | 0.00 | 0.00 | 0.03 | 0.00 | 0.00 | 0.00 |
| 6 | 0.910 | 5.208 | 0.00 | | 0.00 | 0.00 | 0.00 | 0.00 | 0.00 | 0.01 | 0.00 | 0.00 | 0.00 |
| 7 | 0.873 | 5.315 | 0.00 | | 0.00 | 0.00 | 0.00 | 0.00 | 0.00 | 0.03 | 0.00 | 0.00 | 0.00 |
| 8 | 0.813 | 5.508 | 0.00 | | 0.00 | 0.00 | 0.01 | 0.00 | 0.00 | 0.06 | 0.00 | 0.00 | 0.00 |
| 9 | 0.667 | 6.084 | 0.00 | | 0.00 | 0.00 | 0.00 | 0.00 | 0.00 | 0.00 | 0.00 | 0.00 | 0.00 |
| 10 | 0.652 | 6.153 | 0.00 | | 0.00 | 0.00 | 0.06 | 0.00 | 0.04 | 0.30 | 0.00 | 0.00 | 0.00 |
| 11 | 0.618 | 6.317 | 0.00 | | 0.00 | 0.00 | 0.03 | 0.00 | 0.05 | 0.00 | 0.00 | 0.00 | 0.00 |
| 12 | 0.574 | 6.557 | 0.00 | | 0.00 | 0.00 | 0.02 | 0.00 | 0.01 | 0.05 | 0.00 | 0.00 | 0.00 |
| 13 | 0.507 | 6.979 | 0.00 | | 0.00 | 0.00 | 0.00 | 0.00 | 0.01 | 0.20 | 0.00 | 0.00 | 0.00 |
| 14 | 0.474 | 7.217 | 0.00 | | 0.00 | 0.00 | 0.02 | 0.00 | 0.09 | 0.14 | 0.00 | 0.00 | 0.01 |
| 15 | 0.349 | 8.407 | 0.00 | | 0.00 | 0.00 | 0.08 | 0.00 | 0.36 | 0.03 | 0.00 | 0.02 | 0.01 |
| 16 | 0.343 | 8.478 | 0.00 | | 0.00 | 0.00 | 0.10 | 0.00 | 0.25 | 0.02 | 0.00 | 0.00 | 0.00 |
| 17 | 0.270 | 9.559 | 0.00 | | 0.16 | 0.00 | 0.01 | 0.00 | 0.03 | 0.00 | 0.00 | 0.01 | 0.00 |
| 18 | 0.259 | 9.761 | 0.00 | | 0.01 | 0.01 | 0.03 | 0.00 | 0.01 | 0.00 | 0.00 | 0.01 | 0.01 |
| 19 | 0.230 | 10.350 | 0.00 | | 0.02 | 0.01 | 0.01 | 0.00 | 0.00 | 0.00 | 0.01 | 0.10 | 0.01 |
| 20 | 0.213 | 10.755 | 0.00 | | 0.02 | 0.00 | 0.00 | 0.00 | 0.00 | 0.00 | 0.00 | 0.01 | 0.00 |
| 21 | 0.191 | 11.363 | 0.00 | | 0.00 | 0.02 | 0.04 | 0.00 | 0.00 | 0.00 | 0.00 | 0.00 | 0.01 |
| 22 | 0.184 | 11.571 | 0.00 | | 0.01 | 0.00 | 0.32 | 0.00 | 0.04 | 0.00 | 0.00 | 0.02 | 0.03 |
| 23 | 0.176 | 11.855 | 0.00 | | 0.02 | 0.00 | 0.05 | 0.00 | 0.04 | 0.00 | 0.00 | 0.01 | 0.02 |
| 24 | 0.170 | 12.036 | 0.00 | | 0.35 | 0.02 | 0.04 | 0.00 | 0.01 | 0.00 | 0.00 | 0.00 | 0.12 |
| 25 | 0.143 | 13.125 | 0.00 | | 0.01 | 0.00 | 0.00 | 0.00 | 0.00 | 0.00 | 0.00 | 0.00 | 0.29 |
| 26 | 0.135 | 13.520 | 0.00 | | 0.09 | 0.11 | 0.04 | 0.00 | 0.00 | 0.00 | 0.00 | 0.00 | 0.03 |
| 27 | 0.129 | 13.834 | 0.00 | | 0.08 | 0.00 | 0.00 | 0.00 | 0.00 | 0.00 | 0.00 | 0.06 | 0.12 |
| 28 | 0.123 | 14.155 | 0.00 | | 0.00 | 0.10 | 0.06 | 0.00 | 0.01 | 0.00 | 0.00 | 0.00 | 0.17 |
| 29 | 0.106 | 15.255 | 0.00 | | 0.01 | 0.00 | 0.00 | 0.00 | 0.00 | 0.00 | 0.00 | 0.00 | 0.01 |
| 30 | 0.102 | 15.588 | 0.00 | | 0.00 | 0.05 | 0.02 | 0.01 | 0.00 | 0.00 | 0.00 | 0.03 | 0.03 |
| 31 | 0.096 | 16.053 | 0.00 | | 0.00 | **0.56** | 0.01 | 0.00 | 0.00 | 0.00 | 0.00 | 0.02 | 0.00 |
| 32 | 0.084 | 17.139 | 0.00 | | 0.09 | 0.00 | 0.00 | 0.06 | 0.00 | 0.00 | 0.00 | 0.00 | 0.09 |
| 33 | 0.068 | 19.032 | 0.00 | | 0.03 | 0.05 | 0.00 | 0.00 | 0.00 | 0.00 | 0.00 | 0.00 | 0.00 |
| 34 | 0.063 | 19.740 | 0.00 | | 0.01 | 0.00 | 0.00 | **0.67** | 0.00 | 0.00 | 0.00 | 0.00 | 0.00 |
| 35 | 0.043 | 23.954 | 0.00 | | 0.00 | 0.04 | 0.00 | 0.19 | 0.00 | 0.00 | 0.02 | 0.03 | 0.01 |
| 36 | 0.032 | 27.567 | 0.00 | | 0.01 | 0.00 | 0.01 | 0.00 | 0.00 | 0.00 | **0.92** | **0.65** | 0.00 |
| 37 | 0.026 | **31.043** | 0.00 | | 0.00 | 0.00 | 0.00 | 0.00 | 0.00 | 0.00 | 0.01 | 0.00 | 0.01 |
| 38 | 0.023 | **32.454** | 0.00 | | 0.01 | 0.00 | 0.00 | 0.00 | 0.00 | 0.00 | 0.00 | 0.00 | 0.00 |
| 39 | 0.016 | **39.435** | 0.00 | | 0.01 | 0.00 | 0.00 | 0.00 | 0.00 | 0.00 | 0.00 | 0.00 | 0.00 |
| 40 | 0.003 | **87.646** | 1.00 | | 0.05 | 0.01 | 0.00 | 0.05 | 0.00 | 0.00 | 0.01 | 0.00 | 0.01 |

**Table S4** Collinearity diagnostics for the preditors of subjective well-being (*continued*)

| Dimension | Variance proportions | | | | | | | | | | | |
| --- | --- | --- | --- | --- | --- | --- | --- | --- | --- | --- | --- | --- |
|  | *X*_9_ | *X*_10_ | *X*_11_ | *X*_12_ | *X*_13_ | *X*_14_ | *X*_16_ | *X*_17_ | *X*_18_ | *X*_19_ | *X*_20_ | *X*_21_ |
| 1 | 0.00 | 0.00 | 0.00 | 0.00 | 0.00 | 0.00 | 0.00 | 0.00 | 0.00 | 0.00 | 0.00 | 0.00 |
| 2 | 0.00 | 0.00 | 0.00 | 0.00 | 0.00 | 0.00 | 0.00 | 0.00 | 0.00 | 0.02 | 0.03 | 0.04 |
| 3 | 0.00 | 0.01 | 0.00 | 0.01 | 0.00 | 0.00 | 0.00 | 0.00 | 0.00 | 0.01 | 0.01 | 0.01 |
| 4 | 0.00 | 0.04 | 0.00 | 0.01 | 0.00 | 0.00 | 0.00 | 0.00 | 0.00 | 0.02 | 0.00 | 0.01 |
| 5 | 0.00 | 0.08 | 0.00 | 0.01 | 0.00 | 0.00 | 0.00 | 0.00 | 0.00 | 0.01 | 0.06 | 0.00 |
| 6 | 0.00 | 0.00 | 0.00 | 0.00 | 0.00 | 0.00 | 0.00 | 0.00 | 0.00 | 0.00 | 0.46 | 0.01 |
| 7 | 0.00 | 0.03 | 0.00 | 0.02 | 0.00 | 0.00 | 0.00 | 0.00 | 0.00 | 0.05 | 0.17 | 0.00 |
| 8 | 0.00 | 0.01 | 0.00 | 0.01 | 0.00 | 0.00 | 0.00 | 0.00 | 0.00 | 0.03 | 0.16 | 0.17 |
| 9 | 0.00 | 0.03 | 0.00 | 0.01 | 0.00 | 0.00 | 0.00 | 0.00 | 0.00 | 0.32 | 0.00 | 0.22 |
| 10 | 0.00 | 0.06 | 0.00 | 0.05 | 0.00 | 0.00 | 0.00 | 0.00 | 0.00 | 0.01 | 0.00 | 0.07 |
| 11 | 0.00 | 0.00 | 0.00 | 0.13 | 0.00 | 0.00 | 0.00 | 0.00 | 0.00 | 0.07 | 0.00 | 0.02 |
| 12 | 0.00 | 0.01 | 0.00 | **0.64** | 0.00 | 0.00 | 0.00 | 0.00 | 0.00 | 0.00 | 0.00 | 0.00 |
| 13 | 0.00 | 0.01 | 0.00 | 0.01 | 0.00 | 0.00 | 0.00 | 0.00 | 0.00 | 0.01 | 0.00 | 0.00 |
| 14 | 0.00 | 0.00 | 0.00 | 0.01 | 0.00 | 0.00 | 0.00 | 0.00 | 0.00 | 0.00 | 0.00 | 0.00 |
| 15 | 0.00 | 0.00 | 0.00 | 0.00 | 0.00 | 0.00 | 0.00 | 0.00 | 0.00 | 0.00 | 0.00 | 0.00 |
| 16 | 0.00 | 0.00 | 0.00 | 0.00 | 0.01 | 0.00 | 0.00 | 0.00 | 0.00 | 0.00 | 0.00 | 0.00 |
| 17 | 0.00 | 0.01 | 0.01 | 0.00 | 0.07 | 0.01 | 0.00 | 0.00 | 0.00 | 0.00 | 0.00 | 0.00 |
| 18 | 0.00 | 0.03 | 0.17 | 0.02 | 0.09 | 0.01 | 0.00 | 0.00 | 0.00 | 0.00 | 0.00 | 0.00 |
| 19 | 0.00 | 0.00 | 0.00 | 0.01 | 0.01 | 0.00 | 0.00 | 0.00 | 0.00 | 0.01 | 0.00 | 0.03 |
| 20 | 0.00 | 0.00 | 0.08 | 0.00 | 0.01 | 0.00 | 0.00 | 0.00 | 0.00 | 0.19 | 0.07 | 0.31 |
| 21 | 0.00 | 0.00 | 0.10 | 0.01 | 0.02 | 0.01 | 0.00 | 0.00 | 0.00 | 0.02 | 0.00 | 0.02 |
| 22 | 0.00 | 0.00 | 0.09 | 0.00 | 0.04 | 0.00 | 0.00 | 0.00 | 0.00 | 0.00 | 0.00 | 0.02 |
| 23 | 0.00 | 0.00 | 0.37 | 0.00 | 0.01 | 0.00 | 0.00 | 0.00 | 0.00 | 0.02 | 0.01 | 0.04 |
| 24 | 0.00 | 0.03 | 0.00 | 0.01 | 0.05 | 0.04 | 0.00 | 0.00 | 0.00 | 0.00 | 0.00 | 0.00 |
| 25 | 0.00 | 0.00 | 0.06 | 0.00 | 0.16 | 0.00 | 0.00 | 0.01 | 0.00 | 0.01 | 0.00 | 0.01 |
| 26 | 0.00 | 0.00 | 0.00 | 0.00 | 0.14 | 0.00 | 0.00 | 0.01 | 0.00 | 0.01 | 0.00 | 0.00 |
| 27 | 0.00 | 0.00 | 0.04 | 0.00 | 0.05 | 0.05 | 0.00 | 0.07 | 0.00 | 0.01 | 0.00 | 0.00 |
| 28 | 0.00 | 0.00 | 0.01 | 0.01 | 0.17 | 0.14 | 0.00 | 0.00 | 0.00 | 0.00 | 0.00 | 0.00 |
| 29 | 0.00 | 0.00 | 0.00 | 0.00 | 0.07 | 0.32 | 0.00 | 0.03 | 0.00 | 0.00 | 0.00 | 0.00 |
| 30 | 0.00 | 0.00 | 0.02 | 0.00 | 0.01 | 0.01 | 0.00 | 0.33 | 0.00 | 0.05 | 0.00 | 0.00 |
| 31 | 0.00 | 0.00 | 0.00 | 0.01 | 0.00 | 0.28 | 0.00 | 0.02 | 0.00 | 0.00 | 0.00 | 0.00 |
| 32 | 0.00 | 0.00 | 0.03 | 0.01 | 0.00 | 0.04 | 0.01 | 0.35 | 0.00 | 0.02 | 0.00 | 0.00 |
| 33 | 0.00 | 0.01 | 0.00 | 0.00 | 0.02 | 0.00 | 0.00 | 0.01 | 0.00 | 0.00 | 0.00 | 0.00 |
| 34 | 0.00 | 0.01 | 0.00 | 0.00 | 0.02 | 0.03 | 0.00 | 0.00 | 0.00 | 0.00 | 0.00 | 0.00 |
| 35 | 0.12 | 0.09 | 0.00 | 0.01 | 0.04 | 0.02 | 0.08 | 0.01 | 0.05 | 0.01 | 0.00 | 0.00 |
| 36 | 0.02 | 0.01 | 0.00 | 0.00 | 0.00 | 0.00 | 0.00 | 0.00 | 0.00 | 0.00 | 0.00 | 0.00 |
| 37 | 0.46 | 0.25 | 0.01 | 0.00 | 0.00 | 0.00 | 0.01 | 0.00 | 0.00 | 0.00 | 0.00 | 0.00 |
| 38 | 0.27 | 0.18 | 0.00 | 0.00 | 0.00 | 0.00 | 0.21 | 0.00 | 0.11 | 0.02 | 0.00 | 0.00 |
| 39 | 0.00 | 0.00 | 0.00 | 0.00 | 0.00 | 0.00 | 0.48 | 0.06 | **0.63** | 0.05 | 0.00 | 0.00 |
| 40 | 0.12 | 0.07 | 0.01 | 0.00 | 0.01 | 0.01 | 0.20 | 0.08 | 0.20 | 0.03 | 0.00 | 0.00 |

**Table S4** Collinearity diagnostics for the preditors of subjective well-being (*continued*)

| Dimension | Variance proportions | | | | | | | | |
| --- | --- | --- | --- | --- | --- | --- | --- | --- | --- |
|  | *X*_22_ | *X*_23_ | *X*_24_ | *X*_25_ | *X*_26_ | *X*_27_ | *X*_28_ | *X*_29_ | *X*_30_ |
| 1 | 0.00 | 0.00 | 0.00 | 0.00 | 0.00 | 0.00 | 0.00 | 0.00 | 0.00 |
| 2 | 0.03 | 0.01 | 0.00 | 0.04 | 0.00 | 0.00 | 0.00 | 0.00 | 0.00 |
| 3 | 0.02 | 0.00 | 0.00 | 0.02 | 0.00 | 0.00 | 0.00 | 0.00 | 0.00 |
| 4 | 0.01 | 0.00 | **0.68** | 0.00 | 0.00 | 0.00 | 0.00 | 0.00 | 0.00 |
| 5 | 0.20 | 0.00 | 0.25 | 0.00 | 0.00 | 0.00 | 0.00 | 0.00 | 0.00 |
| 6 | 0.00 | 0.35 | 0.01 | 0.00 | 0.00 | 0.00 | 0.00 | 0.00 | 0.00 |
| 7 | 0.23 | 0.18 | 0.04 | 0.00 | 0.00 | 0.00 | 0.00 | 0.00 | 0.00 |
| 8 | 0.19 | 0.14 | 0.00 | 0.00 | 0.00 | 0.00 | 0.00 | 0.00 | 0.00 |
| 9 | 0.06 | 0.03 | 0.00 | 0.00 | 0.00 | 0.00 | 0.00 | 0.00 | 0.00 |
| 10 | 0.04 | 0.04 | 0.00 | 0.00 | 0.00 | 0.00 | 0.00 | 0.00 | 0.00 |
| 11 | 0.00 | 0.01 | 0.00 | 0.00 | 0.00 | 0.00 | 0.00 | 0.00 | 0.03 |
| 12 | 0.00 | 0.00 | 0.00 | 0.00 | 0.00 | 0.00 | 0.00 | 0.00 | 0.01 |
| 13 | 0.00 | 0.00 | 0.00 | 0.00 | 0.00 | 0.00 | 0.00 | 0.00 | 0.00 |
| 14 | 0.00 | 0.00 | 0.00 | 0.00 | 0.00 | 0.00 | 0.00 | 0.00 | 0.01 |
| 15 | 0.00 | 0.00 | 0.00 | 0.00 | 0.00 | 0.01 | 0.01 | 0.00 | 0.00 |
| 16 | 0.00 | 0.00 | 0.00 | 0.00 | 0.00 | 0.02 | 0.03 | 0.00 | 0.00 |
| 17 | 0.00 | 0.00 | 0.00 | 0.01 | 0.00 | 0.01 | 0.02 | 0.00 | 0.00 |
| 18 | 0.00 | 0.00 | 0.00 | 0.00 | 0.00 | 0.00 | 0.00 | 0.09 | 0.00 |
| 19 | 0.01 | 0.02 | 0.00 | 0.09 | 0.00 | 0.00 | 0.02 | 0.01 | 0.01 |
| 20 | 0.15 | 0.14 | 0.01 | **0.69** | 0.00 | 0.01 | 0.01 | 0.00 | 0.00 |
| 21 | 0.01 | 0.01 | 0.00 | 0.03 | 0.00 | 0.01 | 0.04 | 0.04 | 0.01 |
| 22 | 0.00 | 0.01 | 0.00 | 0.02 | 0.00 | 0.06 | 0.35 | 0.01 | 0.02 |
| 23 | 0.02 | 0.03 | 0.00 | 0.06 | 0.00 | 0.00 | 0.05 | 0.37 | 0.00 |
| 24 | 0.00 | 0.00 | 0.00 | 0.00 | 0.00 | 0.02 | 0.03 | 0.01 | 0.01 |
| 25 | 0.00 | 0.01 | 0.00 | 0.01 | 0.00 | 0.05 | 0.07 | 0.31 | 0.00 |
| 26 | 0.00 | 0.00 | 0.00 | 0.00 | 0.00 | 0.32 | 0.23 | 0.00 | 0.09 |
| 27 | 0.00 | 0.00 | 0.00 | 0.00 | 0.00 | 0.18 | 0.02 | 0.00 | 0.19 |
| 28 | 0.00 | 0.00 | 0.00 | 0.00 | 0.00 | 0.20 | 0.09 | 0.02 | 0.13 |
| 29 | 0.00 | 0.00 | 0.00 | 0.00 | 0.00 | 0.00 | 0.00 | 0.00 | 0.02 |
| 30 | 0.00 | 0.00 | 0.00 | 0.00 | 0.00 | 0.03 | 0.00 | 0.01 | 0.25 |
| 31 | 0.00 | 0.00 | 0.00 | 0.00 | 0.00 | 0.03 | 0.01 | 0.00 | 0.05 |
| 32 | 0.00 | 0.00 | 0.00 | 0.00 | 0.01 | 0.01 | 0.01 | 0.05 | 0.03 |
| 33 | 0.00 | 0.00 | 0.00 | 0.00 | 0.00 | 0.00 | 0.00 | 0.05 | 0.02 |
| 34 | 0.00 | 0.00 | 0.00 | 0.00 | 0.00 | 0.00 | 0.00 | 0.01 | 0.00 |
| 35 | 0.00 | 0.00 | 0.00 | 0.00 | 0.04 | 0.00 | 0.00 | 0.01 | 0.02 |
| 36 | 0.00 | 0.00 | 0.00 | 0.00 | 0.00 | 0.00 | 0.00 | 0.00 | 0.04 |
| 37 | 0.00 | 0.00 | 0.00 | 0.00 | **0.50** | 0.00 | 0.00 | 0.00 | 0.00 |
| 38 | 0.00 | 0.00 | 0.00 | 0.00 | 0.37 | 0.00 | 0.00 | 0.00 | 0.01 |
| 39 | 0.00 | 0.00 | 0.00 | 0.00 | 0.00 | 0.00 | 0.00 | 0.00 | 0.00 |
| 40 | 0.00 | 0.00 | 0.00 | 0.00 | 0.09 | 0.01 | 0.00 | 0.00 | 0.02 |

**Table S4** Collinearity diagnostics for the preditors of subjective well-being (*continued*)

| Dimension | Variance proportions | | | | | | | | |
| --- | --- | --- | --- | --- | --- | --- | --- | --- | --- |
|  | *X*_31_ | *X*_32_ | *X*_33_ | *X*_34_ | *X*_35_ | *X*_36_ | *X*_37_ | *X*_38_ | *X*_39_ |
| 1 | 0.00 | 0.00 | 0.00 | 0.00 | 0.00 | 0.00 | 0.00 | 0.00 | 0.00 |
| 2 | 0.00 | 0.00 | 0.00 | 0.00 | 0.00 | 0.00 | 0.02 | 0.00 | 0.00 |
| 3 | 0.00 | 0.00 | 0.00 | 0.00 | 0.00 | 0.00 | 0.05 | 0.00 | 0.00 |
| 4 | 0.00 | 0.00 | 0.00 | 0.00 | 0.00 | 0.00 | 0.00 | 0.00 | 0.00 |
| 5 | 0.00 | 0.00 | 0.00 | 0.00 | 0.00 | 0.00 | 0.01 | 0.00 | 0.00 |
| 6 | 0.00 | 0.00 | 0.00 | 0.00 | 0.00 | 0.00 | 0.00 | 0.00 | 0.00 |
| 7 | 0.00 | 0.00 | 0.00 | 0.00 | 0.00 | 0.00 | 0.00 | 0.00 | 0.00 |
| 8 | 0.00 | 0.00 | 0.00 | 0.00 | 0.00 | 0.00 | 0.01 | 0.00 | 0.00 |
| 9 | 0.01 | 0.00 | 0.00 | 0.00 | 0.04 | 0.00 | 0.00 | 0.00 | 0.00 |
| 10 | 0.00 | 0.00 | 0.00 | 0.00 | 0.08 | 0.00 | 0.01 | 0.00 | 0.00 |
| 11 | 0.13 | 0.00 | 0.00 | 0.00 | 0.05 | 0.00 | 0.01 | 0.03 | 0.00 |
| 12 | 0.06 | 0.00 | 0.00 | 0.00 | 0.02 | 0.00 | 0.01 | 0.01 | 0.00 |
| 13 | 0.06 | 0.00 | 0.00 | 0.01 | 0.44 | 0.00 | 0.16 | 0.00 | 0.00 |
| 14 | 0.08 | 0.00 | 0.00 | 0.00 | 0.07 | 0.00 | **0.51** | 0.01 | 0.00 |
| 15 | 0.03 | 0.18 | 0.02 | 0.01 | 0.00 | 0.00 | 0.00 | 0.01 | 0.00 |
| 16 | 0.00 | 0.30 | 0.03 | 0.00 | 0.03 | 0.00 | 0.00 | 0.00 | 0.00 |
| 17 | 0.04 | 0.03 | 0.00 | 0.07 | 0.00 | 0.00 | 0.00 | 0.23 | 0.00 |
| 18 | 0.03 | 0.02 | 0.01 | 0.04 | 0.00 | 0.02 | 0.07 | 0.05 | 0.00 |
| 19 | 0.04 | 0.00 | 0.00 | 0.00 | 0.02 | 0.00 | 0.02 | 0.36 | 0.00 |
| 20 | 0.00 | 0.00 | 0.00 | 0.00 | 0.00 | 0.00 | 0.00 | 0.03 | 0.00 |
| 21 | 0.03 | 0.01 | 0.00 | **0.54** | 0.11 | 0.00 | 0.01 | 0.07 | 0.00 |
| 22 | 0.02 | 0.00 | 0.00 | 0.06 | 0.00 | 0.00 | 0.00 | 0.02 | 0.00 |
| 23 | 0.00 | 0.00 | 0.00 | 0.00 | 0.00 | 0.01 | 0.02 | 0.00 | 0.00 |
| 24 | 0.04 | 0.00 | 0.00 | 0.13 | 0.06 | 0.02 | 0.01 | 0.00 | 0.00 |
| 25 | 0.00 | 0.01 | 0.01 | 0.03 | 0.01 | 0.00 | 0.01 | 0.03 | 0.01 |
| 26 | 0.03 | 0.00 | 0.01 | 0.00 | 0.01 | 0.00 | 0.00 | 0.03 | 0.00 |
| 27 | 0.05 | 0.02 | 0.01 | 0.05 | 0.00 | 0.01 | 0.01 | 0.03 | 0.01 |
| 28 | 0.08 | 0.02 | 0.02 | 0.01 | 0.01 | 0.00 | 0.02 | 0.02 | 0.00 |
| 29 | 0.01 | 0.27 | **0.52** | 0.00 | 0.00 | 0.00 | 0.00 | 0.00 | 0.00 |
| 30 | 0.12 | 0.06 | 0.15 | 0.01 | 0.00 | 0.00 | 0.00 | 0.01 | 0.03 |
| 31 | 0.01 | 0.07 | 0.17 | 0.00 | 0.01 | 0.01 | 0.00 | 0.00 | 0.00 |
| 32 | 0.03 | 0.00 | 0.01 | 0.01 | 0.00 | 0.10 | 0.00 | 0.00 | 0.12 |
| 33 | 0.00 | 0.00 | 0.00 | 0.00 | 0.00 | 0.48 | 0.01 | 0.00 | 0.46 |
| 34 | 0.00 | 0.00 | 0.00 | 0.00 | 0.00 | 0.14 | 0.00 | 0.01 | 0.15 |
| 35 | 0.04 | 0.00 | 0.01 | 0.01 | 0.00 | 0.11 | 0.00 | 0.03 | 0.15 |
| 36 | 0.01 | 0.00 | 0.00 | 0.01 | 0.00 | 0.01 | 0.00 | 0.00 | 0.00 |
| 37 | 0.00 | 0.00 | 0.00 | 0.00 | 0.00 | 0.06 | 0.00 | 0.00 | 0.03 |
| 38 | 0.00 | 0.00 | 0.00 | 0.00 | 0.00 | 0.00 | 0.00 | 0.00 | 0.01 |
| 39 | 0.00 | 0.00 | 0.00 | 0.00 | 0.00 | 0.00 | 0.00 | 0.00 | 0.00 |
| 40 | 0.02 | 0.00 | 0.01 | 0.01 | 0.00 | 0.04 | 0.00 | 0.01 | 0.02 |

Variables: *X*_0_, year of follow-up; *X*_1_, age; *X*_2_, gender; *X*_3_, ethnic group; *X*_4_, education; *X*_5_, primary occupation before retirement; *X*_6_, current marital status; *X*_7_, have been widowed; *X*_8_, place of residence; *X*_9_, activities of daily living score; *X*_10_, activities of daily living disability; *X*_11_, self-rated health; *X*_12_, number of natural teeth; *X*_13_, visual function; *X*_14_, hearing function; *X*_15_, systolic blood pressure; *X*_16_, diastolic blood pressure; *X*_17_, pulse pressure; *X*_18_, heart rate; *X*_19_, hypertension; *X*_20_, diabetes; *X*_21_, heart disease; *X*_22_, cerebrovascular disease; *X*_23_, respiratory disease; *X*_24_, cancer; *X*_25_, comorbidity of self-reported diseases; *X*_26_, food frequency score; *X*_27_, smoking status; *X*_28_, drinking status; *X*_29_, exercise status; *X*_30_, co-residence; *X*_31_, the number of cohabitants; *X*_32_, the number of biological siblings; *X*_33_, siblings at death; *X*_34_, the number of children; *X*_35_, children at death; *X*_36_, leisure activities score; *X*_37_, financial independence; *X*_38_, caregiver when sick; *X*_39_, access to adequate medical service. Systolic blood pressure has been excluded. The possible presence of multicollinearity has been highlighted using bold text.
