# Supplementary material for: Sociodemographic, health-related, and social predictors of subjective well-being among Chinese oldest-old: a national community-based cohort study
Source: BMC Geriatr. 2021 Feb 16;21:124. doi: 10.1186/s12877-021-02071-7 (PMC7885581; doi:10.1186/s12877-021-02071-7)
Supplement: Supplementary file 2 — Additional file 2. Confirmatory factor analysis for assessing the factor structure of subjective well-being scale. [file 12877_2021_2071_MOESM2_ESM.docx]

**Sociodemographic, health-related, and social predictors of** **subjective well-being among Chinese oldest-old: a national community-based cohort study**

Gang Cheng, Yan Yan

Department of Epidemiology and Health Statistics, Xiangya School of Public Health, Central South University, Changsha, Hunan, China

**Confirmatory factor analysis for assessing the factor structure of subjective well-being scale**

We conducted confirmatory factor analysis (CFA) with two and single-factor models using random sample 1, and a second cross-validation CFA with random sample 2. There were no differences between the two subsamples in terms of demographic characteristics and subjective well-being (SWB) items (Table 1). For the two factor model, life satisfaction and positive affect, which were positively related to SWB, were combined into a factor. Negative affect was another factor, which was negatively related to SWB. Using Mplus 7.4, CFA models were conducted using the WLSMV estimator. We used a correlated uniqueness (CU) model of multitrait-multimethod (MTMM) to allow item residuals to correlate based on these item characteristics. For model fit evaluation, root mean square error of approximation less than 0.06, comparative fit index and Tucker-Lewis index more than 0.95, and weighted root mean square residual (WRMR) less than 1.0 indicate a good fit. Table 2 shows a good fit both for the single-factor models using random samples 1 and 2. Approximate fit indices for the two-factor model were good, except for WRMR (1.161). All standardized factor loadings for the two-factor structure were significant and above 0.35 (Table 3). And factor loadings for the single factor structure in samples 1 and 2 were both significant, ranging from 0.33 to 0.61 and 0.34 to 0.59.

Table 1. Demographic characteristics and subjective well-being items for random split-half samples (*N*=30,317)

| Variables | Sample 1 (*n*=14996) | | Sample 2 (*n*=15321) | | *χ*^2^ | *p*-value |
| --- | --- | --- | --- | --- | --- | --- |
|  | *n* | % | *n* | % |  |  |
| **Age group** |  |  |  |  |  |  |
| 80-89 years | 6610 | 44.1 | 6809 | 44.4 | 0.448 | 0.799 |
| 90-99 years | 4931 | 32.9 | 4992 | 32.6 |  |  |
| ≥100 years | 3455 | 23.0 | 3520 | 23.0 |  |  |
| **Gender** |  |  |  |  |  |  |
| Women | 8682 | 57.9 | 8981 | 58.6 | 1.631 | 0.202 |
| Men | 6314 | 42.1 | 6340 | 41.4 |  |  |
| **Ethnic group** |  |  |  |  |  |  |
| Han nationality | 14080 | 93.9 | 14362 | 93.7 | 0.298 | 0.585 |
| Ethnic minorities | 916 | 6.1 | 959 | 6.3 |  |  |
| **Subjective well-being items (answered “very good” or “always”)** | | | | |  |  |
| Life satisfaction | 2472 | 16.5 | 2458 | 16.0 | 2.718 | 0.606 |
| Optimism | 2174 | 14.5 | 2066 | 13.5 | 8.696 | 0.069 |
| Happiness | 2614 | 17.4 | 2712 | 17.7 | 0.586 | 0.965 |
| Personal control | 3750 | 25.0 | 3839 | 25.1 | 1.000 | 0.910 |
| Conscientiousness | 2443 | 16.3 | 2472 | 16.1 | 7.606 | 0.107 |
| Anxiety | 241 | 1.6 | 222 | 1.4 | 3.735 | 0.443 |
| Loneliness | 318 | 2.1 | 302 | 2.0 | 1.803 | 0.772 |
| Uselessness | 1091 | 7.3 | 1150 | 7.5 | 6.144 | 0.189 |

Table 2. Fit indices for confirmatory factor analysis models.

| Fit index | Sample 1 (*n*=14996) | | Sample 2 (*n*=15321) |
| --- | --- | --- | --- |
|  | Two-factor | Single-factor | Single-factor |
| χ^2^(df) | 122.473(8) * | 65.129(2) * | 77.574(2) * |
| RMSEA (90% CI) | 0.031(0.026, 0.036) | 0.046(0.037, 0.056) | 0.050(0.041, 0.059) |
| CFI | 0.997 | 0.998 | 0.998 |
| TLI | 0.989 | 0.976 | 0.973 |
| WRMR | 1.161 | 0.806 | 0.871 |

Abbreviation: RMSEA, root mean square error of approximation; CFI, comparative fit index; TLI, Tucker-Lewis index; WRMR, weighted root mean square residual. Level of significance: * *p* < 0.001.

Table 3. Standardized CFA factor loadings for subjective well-being items.

| Item | Sample 1 (*n*=14996) | | | Sample 2 (*n*=15321) |
| --- | --- | --- | --- | --- |
|  | Two factor | | Single-factor | Single-factor |
| Life satisfaction | 0.639(0.009) |  | 0.549(0.014) | 0.526(0.014) |
| Optimism | 0.705(0.010) |  | 0.605(0.010) | 0.590(0.010) |
| Happiness | 0.511(0.009) |  | 0.572(0.011) | 0.567(0.011) |
| Personal control | 0.348(0.010) |  | 0.327(0.011) | 0.343(0.011) |
| Conscientiousness | 0.534(0.010) |  | 0.439(0.015) | 0.423(0.015) |
| Anxiety |  | 0.650(0.013) | 0.459(0.012) | 0.492(0.012) |
| Loneliness |  | 0.736(0.013) | 0.500(0.010) | 0.503(0.010) |
| Uselessness |  | 0.536(0.009) | 0.473(0.013) | 0.456(0.013) |

Abbreviation: CFA, confirmatory factor analysis. Data are expressed as estimates (standard errors). All factor loadings are significant at *p* < 0.001.
